# SMAX1/SMXL2 regulate root and root hair development downstream of KAI2-mediated signalling in Arabidopsis

**DOI:** 10.1371/journal.pgen.1008327

**Published:** 2019-08-29

**Authors:** José Antonio Villaécija-Aguilar, Maxime Hamon-Josse, Samy Carbonnel, Annika Kretschmar, Christian Schmidt, Corinna Dawid, Tom Bennett, Caroline Gutjahr

**Affiliations:** 1 Faculty of Biology, Genetics, LMU Munich, Biocenter Martinsried, Martinsried, Germany; 2 Plant Genetics, TUM School of Life Sciences Weihenstephan, Technical University of Munich (TUM), Freising, Germany; 3 School of Biology, Faculty of Biological Sciences, University of Leeds, Leeds, United Kingdom; 4 Chair of Food Chemistry and Molecular Sensory Science, TUM School of Life Sciences Weihenstephan, Technical University of Munich (TUM), Freising, Germany; 5 Sainsbury Laboratory Cambridge University, Cambridge, United Kingdom; Wake Forest University, UNITED STATES

## Abstract

Karrikins are smoke-derived compounds presumed to mimic endogenous signalling molecules (KAI2-ligand, KL), whose signalling pathway is closely related to that of strigolactones (SLs), important regulators of plant development. Both karrikins/KLs and SLs are perceived by closely related α/β hydrolase receptors (KAI2 and D14 respectively), and signalling through both receptors requires the F-box protein MAX2. Furthermore, both pathways trigger proteasome-mediated degradation of related SMAX1-LIKE (SMXL) proteins, to influence development. It has previously been suggested in multiple studies that SLs are important regulators of root and root hair development in Arabidopsis, but these conclusions are based on phenotypes observed in the non-specific *max2* mutants and by use of *racemic-GR24*, a mixture of stereoisomers that activates both D14 and KAI2 signalling pathways. Here, we demonstrate that the majority of the effects on Arabidopsis root development previously attributed to SL signalling are actually mediated by the KAI2 signalling pathway. Using mutants defective in SL or KL synthesis and/or perception, we show that KAI2-mediated signalling alone regulates root hair density and root hair length as well as root skewing, straightness and diameter, while both KAI2 and D14 pathways regulate lateral root density and epidermal cell length. We test the key hypothesis that KAI2 signals by a non-canonical receptor-target mechanism in the context of root development. Our results provide no evidence for this, and we instead show that all effects of KAI2 in the root can be explained by canonical SMAX1/SMXL2 activity. However, we do find evidence for non-canonical GR24 ligand-receptor interactions in D14/KAI2-mediated root hair development. Overall, our results demonstrate that the KAI2 signalling pathway is an important new regulator of root hair and root development in Arabidopsis and lay an important basis for research into a molecular understanding of how very similar and partially overlapping hormone signalling pathways regulate different phenotypic outputs.

## Author summary

Karrikins are plant signaling compounds from smoke, which induce germination of fire-following plants. They likely mimic endogenous plant hormones (KAI2-ligand, KL), because Arabidopsis karrikin receptor mutants display shoot developmental phenotypes. Perception of karrikins/KL is very similar to that of another plant hormone class, strigolactones (SLs). Both hormones bind to the related α/β-fold hydrolase receptors KAI2 and D14 respectively, which both interact with the F-box protein MORE AXILLIARY BRANCHES2 (MAX2), for ubiquitylation and subsequent degradation of KL- or SL-signalling specific proteins of the SMXL family. Based on *max2* mutant phenotypes it has been suggested that the development of Arabidopsis root architecture and root hairs is regulated by SL signaling. However, *max2* does not distinguish between the two signalling pathways. We genetically dissected the role of KL and SL signalling in root and root hair development in Arabidopsis seedlings and show that most root traits are regulated by KL and not by SL signaling: lateral root density is controlled by KL and SL signalling together, while root growth direction, root straightness and root hair development are determined by KL signalling alone. Thus, KL signalling regulates vital plant traits for nutrient and water uptake as well as anchorage to the ground.

## Introduction

Plant roots continually integrate environmental information to make decisions about their development, and to optimize their growth for optimal nutrient uptake and anchorage. Increased lateral root formation and root hair growth are necessary to compensate for low nutrient availability in the soil by increasing the root surface area for nutrient uptake, while directional growth is required to avoid stressors such as salt, obstacles or to reach moisture [1–5]. Root development is regulated by a number of phytohormones, low-molecular-weight signalling molecules, which mediate localized developmental responses as well as transmission and integration of information across long distances. Among them, SLs have been suggested to act as important regulators of Arabidopsis seedling root architecture and root hair development [6–9]. However, the exact role of SLs in root development remains uncertain, due to interpretational difficulties inherent in the materials used by those studies, namely *max2* mutants and the synthetic strigolactone *racemic-*GR24 (see below, [10]).

Genes involved in SL biosynthesis have been identified in several plant species [10]. The universal SL precursor carlactone is synthesized from β-carotene by a core pathway of three enzymes; the isomerase DWARF27, and the carotenoid cleavage dioxygenases CCD7 and CCD8 (MAX3 and MAX4 in Arabidopsis) [11]. Carlactone is then modified by a variety of enzymes, including the cytochrome P450s of the MAX1 sub-family, to create a range of active SL molecules [12]. SLs are perceived and hydrolysed by the α/β hydrolase receptor DWARF14 (D14) [13–16]. D14 interacts with the SCF^MAX2^ E3 ubiquitin ligase complex to induce ubiquitylation and subsequent degradation of target proteins, essential to trigger SL signal transduction [15, 17].

A second, closely related signalling pathway also acts through the SCF^MAX2^ complex [18, 19]. In this pathway MAX2 is thought to interact with KAI2 (KARRIKIN-INSENSITIVE2), an α/β hydrolase receptor protein, which is encoded by an evolutionary older paralog of D14 [20–22]. KAI2 was originally identified as a receptor for karrikins, a family of butenolide compounds found in the smoke of burnt plant material [19, 23]. In fire-following species, karrikins are used as germination cues, indicating the removal of competing plants. However, karrikins promote germination in a range of flowering plant species, which do not germinate after fire [24–26] and KAI2 is required for a number of developmental traits in Arabidopsis not related to germination as well as for arbuscular mycorrhiza symbiosis in rice [19, 27–30]. Because of these roles of KAI2, karrikins are thought to mimic the action of a yet unknown endogenous plant signalling molecule, which is currently denoted KAI2-ligand (KL) [31–33].

Since KAI2 and D14 act through the same F-box protein MAX2, *max2* mutants are insensitive to both SLs and karrikins, and display the combined phenotypes of *d14* and *kai2* mutants [18, 19, 27, 28]. Most studies aimed at understanding the role of SLs in Arabidopsis root development have used *max2* mutants—likely for historical reasons because they were available prior to *d14* and *kai2*. However, if only *max2* mutants are employed without comparison with the specific receptor mutants, the root phenotypes cannot be reliably attributed to either SL or KL signalling. The second difficulty in interpreting previously published root phenotypes arises from the experimental use of the strigolactone analog GR24, which in standard preparations is a racemic mix of two stereoisomers (*rac-*GR24). While one stereoisomer (GR24^5DS^) is a potent activator of D14 signalling, the non-natural stereoisomer (GR24^ent-5DS^) appears to stimulate KAI2 signalling [31, 34]. As such, the indiscriminate use of *rac*-GR24 has created a legacy of interpretational problems in previous studies, and incorrect attribution of phenotypic effects to SL signalling [10, 34].

Genetic and biochemical evidence indicates that the D14-SCF^MAX2^ and the KAI2-SCF^MAX2^ complex target a group of regulators–the SMXL (SMAX1-LIKE) family of proteins with weak homology to ClpB type chaperonins–for ubiquitylation and subsequent proteolytic degradation. In Arabidopsis, the genetically defined degradation targets of KL signalling are SMAX1 (SUPPRESSOR OF MAX2 1) and SMXL2, while the targets of SL signalling are SMXL6, SMXL7 and SMXL8 (hereafter SMXL678) [27, 35–37]. In the shoot, the hormone-induced turnover of SMXL678 proteins is key to correctly shaping shoot architecture [38]. The exact molecular function of the SMXL proteins is poorly understood. SMXL678 and their rice ortholog *D53* have been associated with transcriptional regulation, since they physically interact with TOPLESS-RELATED (TPR) co-repressor proteins [27, 39, 40]. Rice D53 interacts with IPA1, a SQUAMOSA PROMOTER-BINDING FAMILY LIKE (SPL) transcription factor in the regulation of shoot branching and may recruit TPR to repress IPA1-mediated transcription [41]. However, they have also been found to be involved in enhancing PIN1 accumulation at the basal membrane of stem xylem parenchyma cells and auxin transport [38]. The role of SMXL proteins in root and root hair development has not been comprehensively addressed. Initial observations suggested mutations of *SMXL678* suppress the enhanced lateral root density phenotype of *max2* [27], while unexpectedly the increased root skewing phenotype, recently described for *kai2* and *max2* mutants was also suppressed by *smxl678* [29]. These data have been used to propose the existence of non-canonical D14/KAI2 signalling cascades in the context of lateral root development and root skewing [10, 29].

In this study, we dissected the roles of SLs and KLs in the control of root development in Arabidopsis. We aimed to test the important hypothesis that root development might be mediated by non-canonical receptor-target interactions between D14, KAI2 and SMAX1/SMXL2, SMXL678. Our results show that KAI2 is much more important than previously realized in the regulation of root development, and that many effects previously attributed to SL signalling are actually mediated by KAI2 (and therefore KL signalling). We find no evidence for non-canonical receptor-target interactions, but conversely find surprising evidence of non-canonical GR24 ligand-receptor interactions in both KAI2 and D14 signalling.

## Results

### Strigolactones have relatively minor effects on seedling root architecture

**Fig 1:**
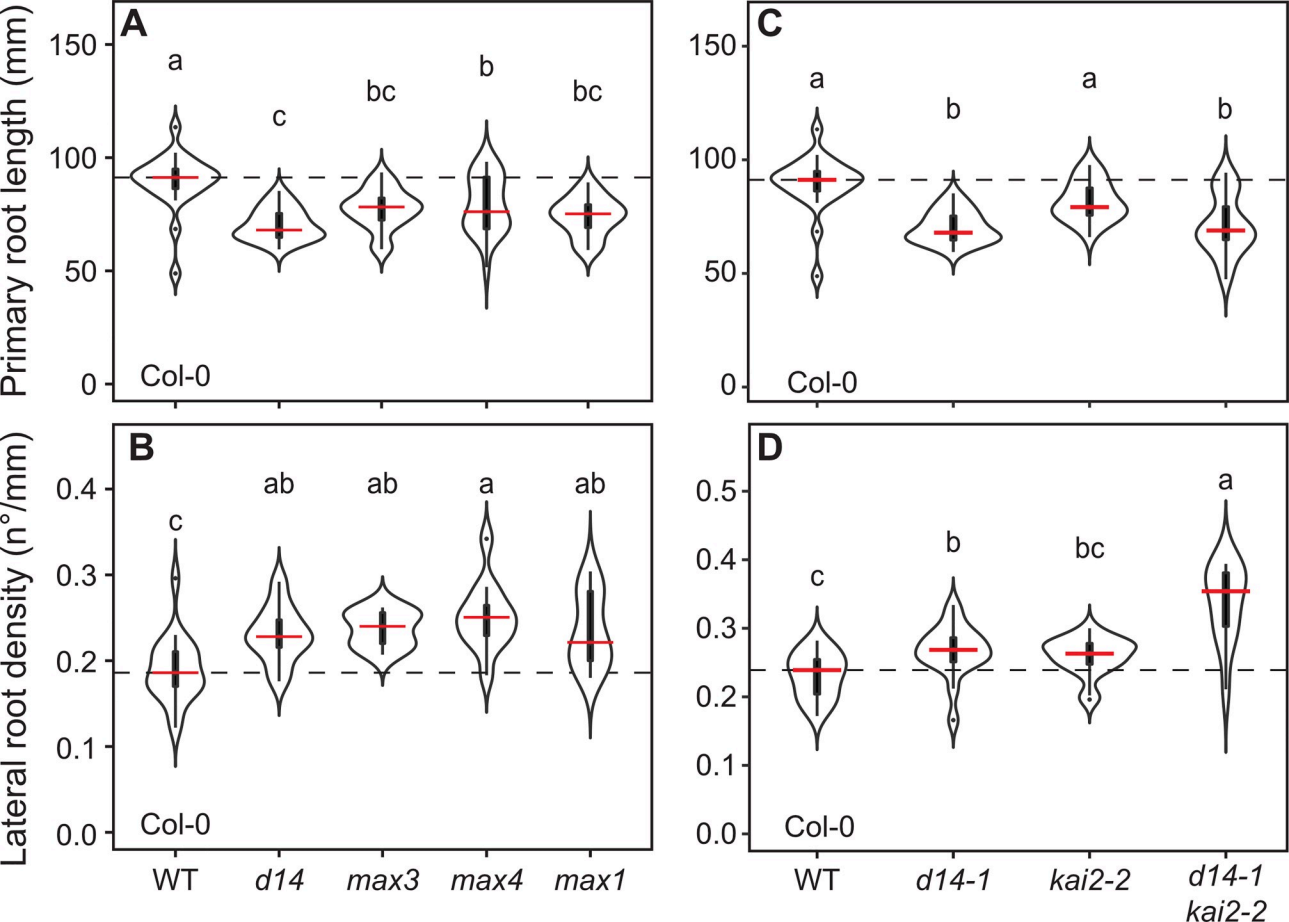
SL and KL signalling additively regulate lateral root density. **(A)** Primary root length (experiment 3 in S1 Fig) and **(B)** lateral root density (experiment 1 in S1 Fig) of Col-0 wild type, the strigolactone perception mutant *d14-1* and the strigolactone biosynthesis mutants *max3-9*, *max4-5* and *max1-1* (arranged in pathway order). **(C)** Primary root length and **(D)** lateral root density in the *d14-1 kai2-2* double mutant and the respective single mutants. Data in **(C)** form part of the same dataset in **(A)**, and PRL for the Col-0 and *d14-1* genotypes are also shown in **(A)**. LRD was recorded at 10 dpg. The outline of the violin plots represents the probability of the kernel density. Black boxes represent interquartile ranges (IQR), with the red horizontal line representing the median; whiskers extend to the highest and lowest data point but no more than ±1.5 times the IQR from the box; outliers are plotted individually. Different letters indicate different statistical groups (ANOVA, posthoc Tukey, p≤ 0.001 **(A)** F_4,111_ = 11.81, n = 19–25 **(B)** F_4,58_ = 5.626, n = 8–18 **(C)** F_3,88_ = 17,83, n = 21–26 **(D)** F_3,63_ = 19.82, n = 11–18).

SLs have previously been described to regulate primary root length (PRL), lateral root density (LRD) and root hair development [6, 8, 9, 42]. We re-assessed the specific roles of SL signalling in root development in mutants specifically affected in SL biosynthesis, namely the SL biosynthesis mutants *max3-9*, *max4-5* and *max1-1* (here arranged in pathway order). Surprisingly, we found that SLs only have subtle effects on root architecture. We observed decreased primary root length (PRL) and increased lateral root density (LRD) in SL biosynthesis mutants across many experiments, but rarely at the same time (summarized in S1 Fig). For instance, Fig 1A shows reduction in PRL relative to Col-0 in all SL biosynthesis mutants, but in the same experiment LRD was not altered (S1 Fig). Conversely, Fig 1B shows increased LRD in SL biosynthesis mutants relative to Col-0, but PRL was not altered in the same experiment (S1 Fig). Thus, consistent with previous reports [8], we found that SL signalling has subtle, and possibly mutually exclusive, effects on PRL and LRD of Arabidopsis, which appear to be sensitive to small differences in growth conditions.

**Fig 2:**
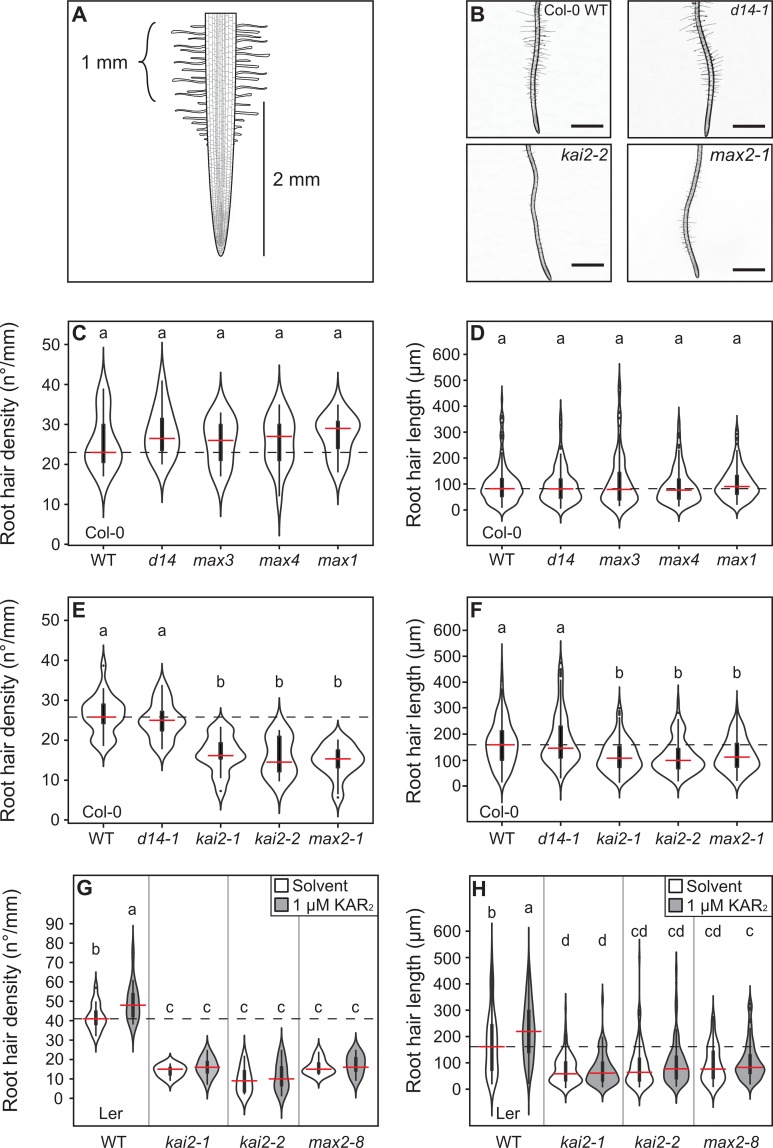
KL perception mutants are impaired in root hair development. **(A)** Diagram showing the primary root zone used for root hair phenotyping (curly bracket). Root hair density and length were quantified in 1 mm primary root length between 2 and 3 mm from the root tip. **(B)** Representative images of root hair phenotypes of the indicated genotypes. Scale bar, 1 mm. **(C,E,G)** Root hair density and **(D,F,G)** root hair length in **(C,D)** Col-0 wild type, the strigolactone perception mutant *d14-1* and the strigolactone biosynthesis mutants *max3-9*, *max4-5* and *max1-1* (arranged in pathway order), **(E,F)** the indicated karrikin perception mutants and **(G,H)** Ler wild type and indicated karrikin perception mutants, treated with solvent (70% Methanol) or 1 μM KAR_2._. The outline of the violin plots represents the probability of the kernel density. Black boxes represent interquartile ranges (IQR), with the red horizontal line representing the median; whiskers extend to the highest and lowest data point but no more than ±1.5 times the IQR from the box; outliers are plotted individually. Different letters indicate different statistical groups (ANOVA, posthoc Tukey, **(C)** F_4,65_ = 0.242, n = 10–18; p≤0.05, **(D)** F_4,718_ = 1.291, n = 10–13, p≤0.05, **(E)** F_4,88_ = 28.9, n = 11–24, p≤0.001), **(F)** F_4,825_ = 23.43, n = 10–13, p≤ 0.001, **(G)** F_7,96_ = 60.79, n = 10–15, p≤ 0.001, **(H)** F_7,975_ = 45.39, n = 10–13, p≤ 0.001).

We also examined root hair formation in the suite of SL biosynthesis mutants. Contrary to previous assumptions [7] we found that neither root hair density (RHD) nor root hair length (RHL) are altered in any of the SL biosynthesis mutants (Fig 2A, 2C and 2D). Thus, the previously observed root hair phenotypes of *max2* mutants must be caused by defects other than SL signalling, for example in KL signalling.

### D14 and KAI2 co-regulate lateral root density

The phenotypes present in SL-specific biosynthesis mutants are insufficient to account for previously described effects of *max2* on root development. We therefore hypothesized that KAI2 signalling may play an important role in the regulation of root and root hair development, and we therefore compared and contrasted root development in *d14* and *kai2* mutants. In the case of LRD, we observed that *d14-1* causes increased LRD and/or reduced PRL, consistent with the phenotypes of SL biosynthesis mutants (Fig 1A–1D). We also observed that two allelic *kai2* mutants (*kai2-1*, *kai2-2*) in the Col-0 background, showed increased LRD of around the same magnitude as *d14-1* (Fig 1D, S2A Fig), with no clear effect on PRL (Fig 1C). This phenotype in *kai2* was particularly evident at 6dpg, and became less evident at later time points. For *d14*, the opposite pattern was seen, and the LRD phenotype only became evident at later time points (Fig 1D, S2B Fig). Thus, at least some of the confusion about the role of these pathways in regulation of lateral root development may result from the staging of experiments. Taken together, our results suggest that both SL and KL signalling regulate LRD in Arabidopsis. We further tested this idea by examining LRD in *d14 kai2* double mutants. The *d14-1 kai2-2* mutant showed a very strong and consistent increase in LRD in comparison to Col-0, *d14-1* and *kai2-2* (Fig 1D, S2B Fig). The increase in LRD was always greater in *d14-1 kai2-2* than in the single mutants (Fig 1D). Thus, both KL and SL signalling regulate LRD in an additive manner, possibly by affecting lateral root development at different developmental stages and time points.

### KAI2 but not D14 regulates root hair development

Given the lack of root hair phenotype in SL biosynthesis mutants, we hypothesized that KAI2 and not D14 signalling would regulate root hair development. Consistent with this hypothesis, we observed no RHD or RHL phenotype in *d14-1* (Fig 2B–2F). Conversely, RHD and RHL were strongly decreased in two allelic *kai2* mutants in Col-0 as well as Ler, and they perfectly phenocopied the root hair phenotype of *max2* mutants (Fig 2B, 2E–2H). Thus, the root hair phenotypes previously observed in *max2* mutants and attributed to the lack of SL signalling are actually caused by a lack of KL signalling. To confirm this, we assessed whether root hair development can be influenced by exogenous addition of karrikin. Treatment with 1 μM KAR_2_ increased RHD and RHL relative to control treatments in a KAI2 and MAX2-dependent manner (Fig 2G and 2H), corroborating the role of KL-signalling in promoting root hair development.

### KAI2 signalling regulates root skewing and waving

**Fig 3:**
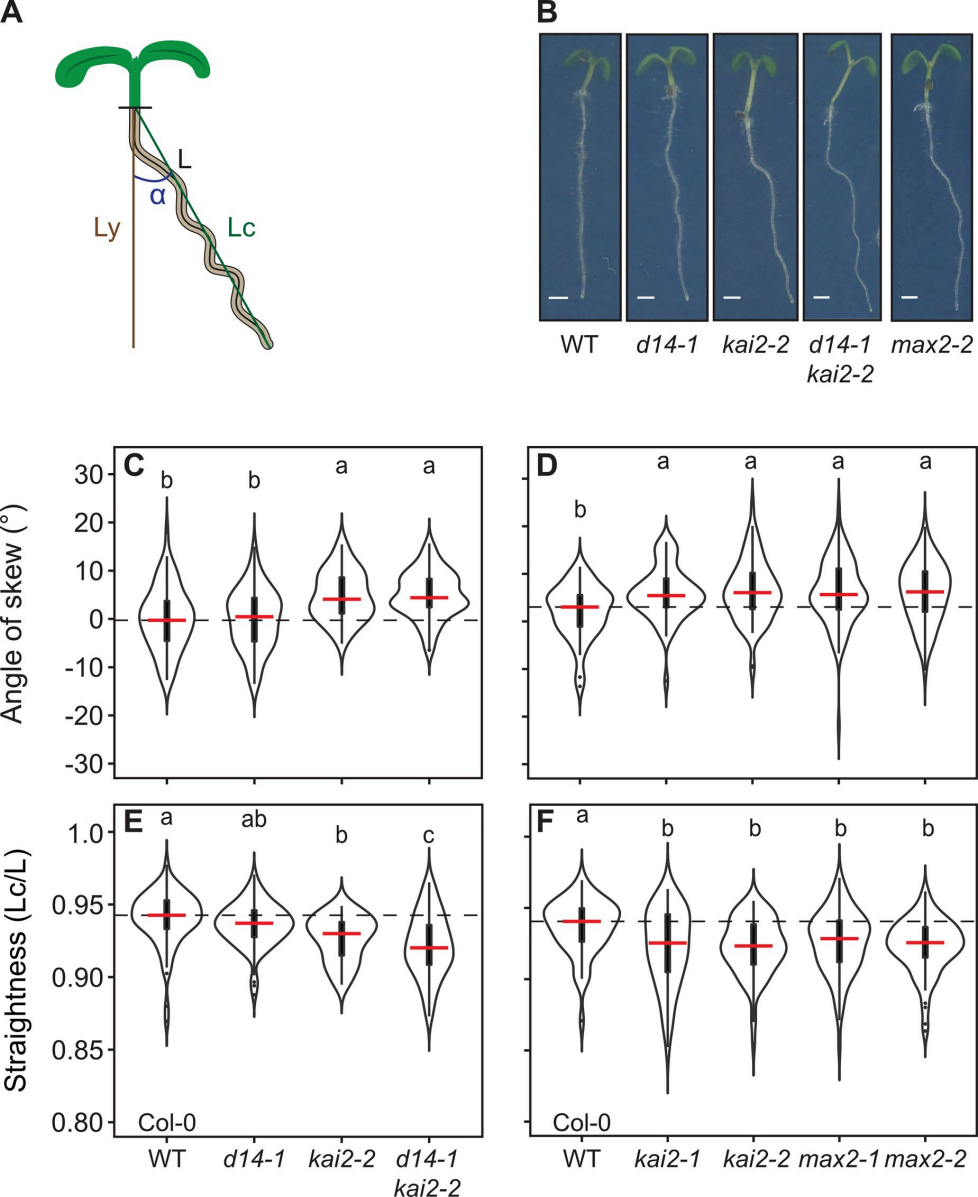
KL perception mutants display exaggerated skewing and waving. **(A)** Diagram showing how skewing-angle and root straightness were determined. Skewing was quantified by measuring the angle between the vertical axis (Ly) defined as 0°, and the root tip. Right or left skewing is indicated by positive or negative values, respectively. Straightness was calculated as the ratio of the straight line between the hypocotyl-root junction and the root tip (green line, Lc) and the total root length (L). **(B)** Images of representative 5-days-old seedlings of the indicated genotypes. Scale bars, 1 mm. **(C**, **D)** Root skewing and **(E** and **F)** root straightness of the indicated genotypes. The outline of the violin plot represents the probability of the kernel density. Black boxes represent interquartile ranges (IQR), the red horizontal line representing the median; whiskers extend to the highest and lowest data point but no more than ±1.5 times the IQR from the box; outliers are plotted individually. Different letters indicate different statistical groups (ANOVA, posthoc Tukey, p≤ 0.001, **(C)** F_3,315_ = 16.08, n > 60 **(D)** F_4,347_ = 4.762, n > 50 **(E)** F_3,315_ = 13.62, n > 60 **(F)** F_4,347_ = 4.28, n > 50).

In addition to lateral root and root hair phenotypes, we observed that *kai2* mutants display increased skewing along the surface of vertically-oriented agar plates, in the Col-0 and in the Ler ecotype (Fig 3A–3D, S3 Fig), consistent with a recent report that described this phenotype in *kai2* mutants in Ler [29]. This right-handed skewing is a well-established effect of growing Arabidopsis roots on the surface of agar plates, and probably arises from a combination of circumnutation and thigmotropic responses [43, 44]. Increased skewing is also observed for *max2* mutants, but not for SL biosynthesis mutants, nor *d14* (Fig 3B and 3C; S3A Fig). The skewing phenotype of the *d14-1 kai2-2* double mutant in the Col-0 background is equal to *kai2-2* (Col-0), confirming that SL perception is not involved in regulating root growth direction (Fig 3C).

The increased skewing in the *kai2* and *max2* mutants is accompanied by increased root waving, which is displayed as a decrease in root ‘straightness’ (Fig 3A, 3E and 3F, S3B Fig). Again, this waving phenotype is not observed in *d14-1* or SL biosynthesis mutants (Fig 3E, S3B Fig). The waving phenotype is separable from the skewing phenotype, and growth on plates inclined at 45° generally increases waving relative to plates grown at 90°, while altering skewing only in the Ler but not in the Col-0 wild type (S3C–S3G Fig).

### KAI2 regulates skewing independently of epidermal cell elongation and root diameter

**Fig 4:**
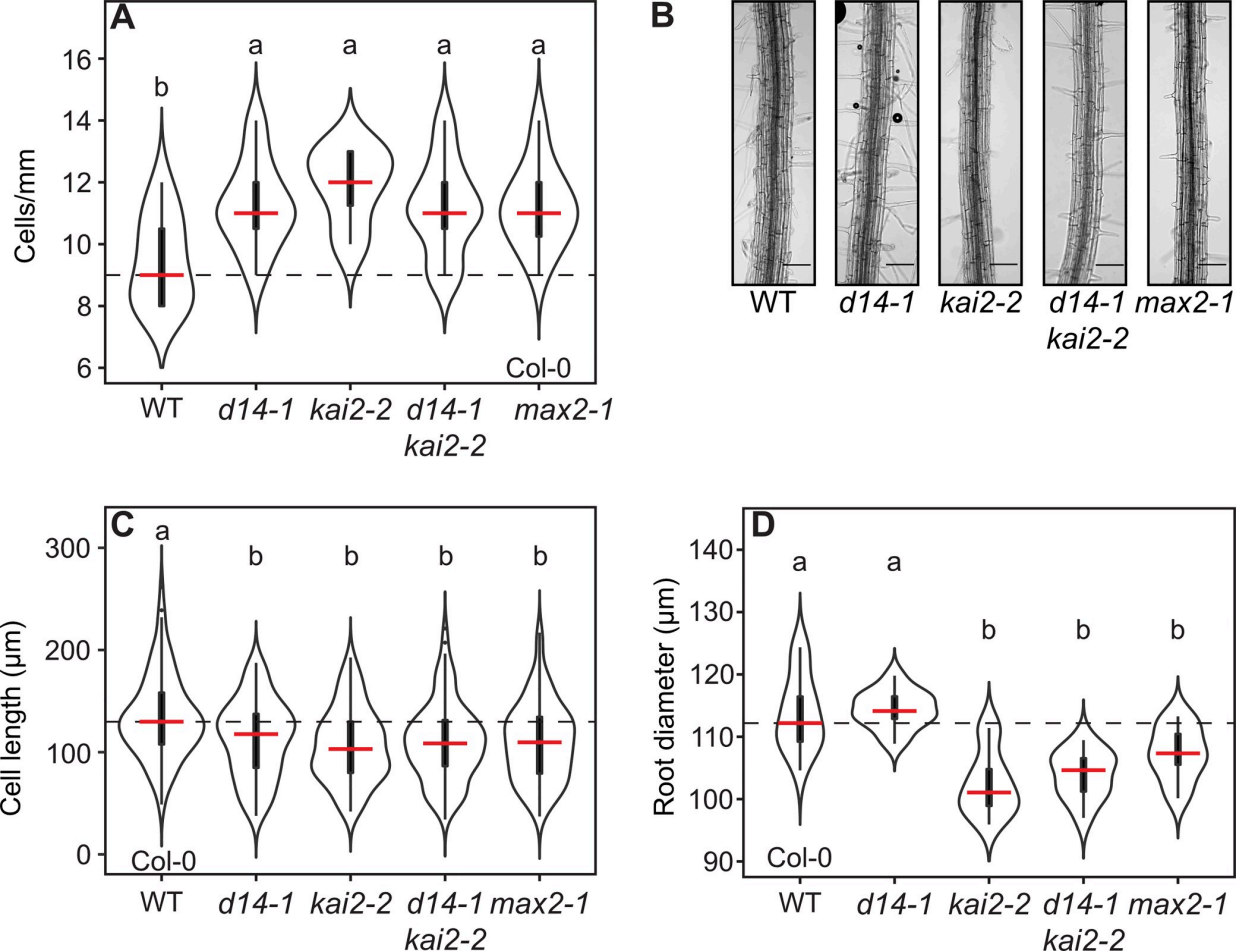
KL perception mutants exhibit decreased epidermal cell lengths and root diameter. **(A)** Number of root epidermal cells per mm of the indicated genotypes. **(B)** Images of representative roots between 2 and 3 mm from the root tip of 5-days-old seedlings of the indicated genotypes. Scale bars, 0.1 mm. **(C)** Root cell length and **(D)** root diameter of the indicated genotypes. The outline of the violin plots represents the probability of the kernel density. Black boxes represent interquartile ranges (IQR), the red horizontal line representing the median; whiskers extend to the highest and lowest data point but no more than ±1.5 times the IQR from the box; outliers are plotted individually. Different letters indicate different statistical groups (ANOVA, posthoc Tukey, **(A)** F_4,52_ = 4.715, n = 9–13, p≤ 0.01, **(C)** F_3,392_ = 10.64, n = 10–11, p≤ 0.001, **(D)** F_4,50_ = 15.95, n = 10–12, p≤0.001).

Skewing is often associated with epidermal cell file rotation [44]. To determine whether skewing of *kai2* and *max2* mutants is associated with cell file rotation [45], we inspected epidermal cells between 2 and 3mm above the root tip in *kai2* mutants. Cell length was reduced in *kai2* and *max2* mutants relative to wild-type in both Col-0 and Ler backgrounds (with a concomitant increase in cells/mm) (Fig 4A and 4C, S4A and S4C Fig). However, a careful microscopic inspection of the root surface of *kai2* and *max2* mutants did not show any signs of epidermal cell file rotation, instead they were clearly vertically orientated (Fig 4B, S4B Fig). This is in contrast to the results of [29], who observed increased cell file rotation in *kai2* and *max2* mutants in Ler at a 45° growth angle. Since at a 90° growth angle we observed a skewing phenotype but no cell file rotation, we conclude that there is likely no connection between any cell file rotation phenotype in KL perception mutants and their skewing phenotype. Interestingly, also the SL perception mutant *d14* displayed the short epidermal cell phenotype but had no skewing phenotype, clearly demonstrating that there is no connection between epidermal cell length and skewing in these receptor mutants (Fig 4A and 4C; S4A and S4C Fig).

**Fig 5:**
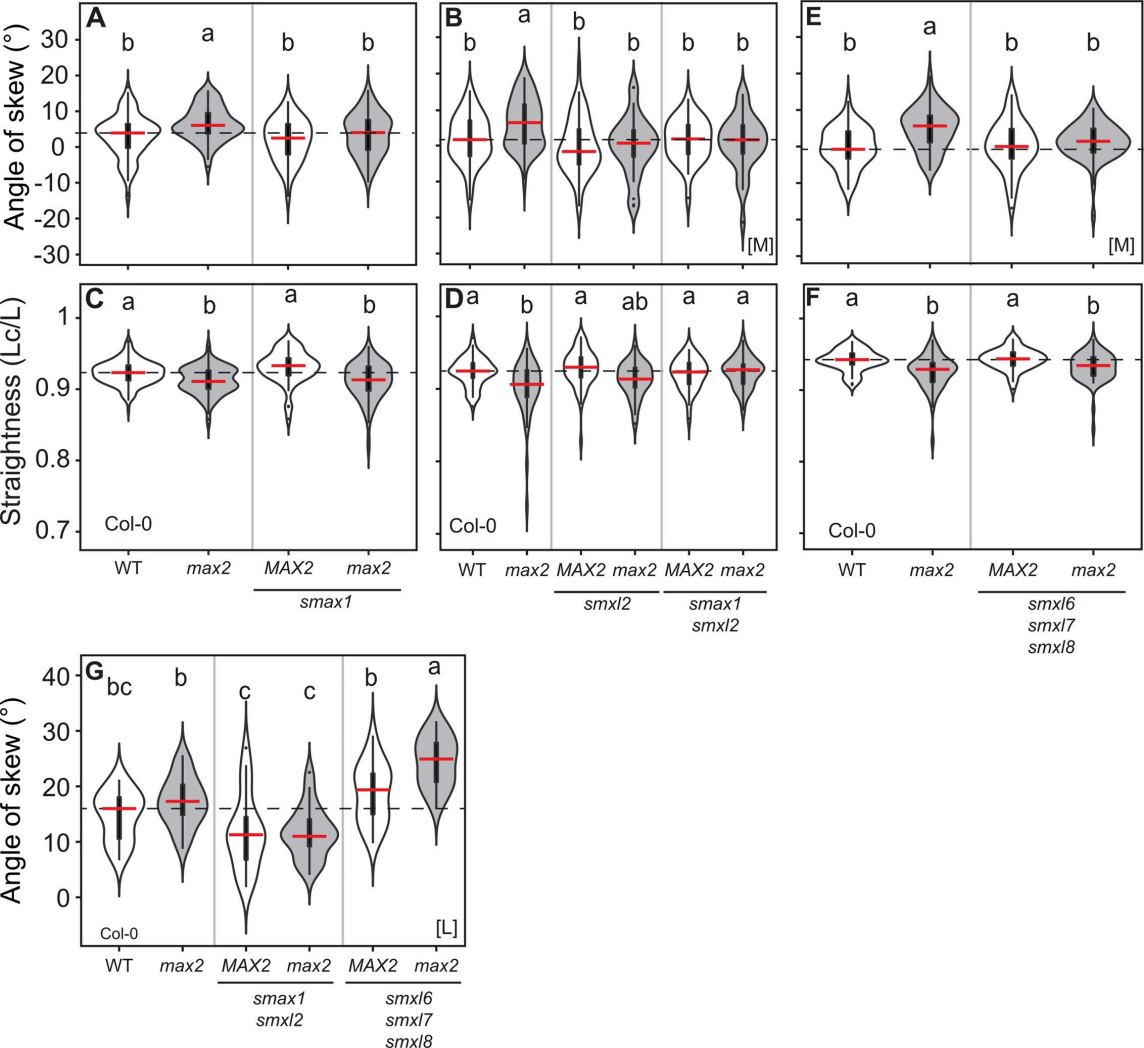
SMAX1 and SMXL2 regulate skewing and root straightness. **(A, B, E, F)** Root skewing and **(C, D, G)** root straightness in Col-0 wild type and the indicated genotypes (the mutant alleles are *max2-1*, *smax1-2*, *smxl2-1*, *smxl6-4*, *smxl7-3* and *smxl8-1*). The outline of the violin plot represents the probability of the kernel density. Black boxes represent interquartile ranges (IQR), with the red horizontal line representing the median; whiskers extend to the highest and lowest data point but no more than ±1.5 times the IQR from the box; outliers are plotted individually. Different letters indicate different statistical groups (ANOVA, posthoc Tukey, p≤0.001 **(A)** F_3,345_ = 7.612, n > 60; **(B)** F_5,259_ = 5.051, n > 30; **(C)** F_3,440_ = 16.32, n > 60; **(D)** F_5,261_ = 6.57, n > 30 **(E)** F_3,209_ = 8.784, n > 45 **(F)** F_3,209_ = 10.22, n > 45; **(G)** F_5,127_ = 21.07, n = 21). [M] = experiment performed in Munich, [L] = experiment performed in Leeds.

It has also been speculated that a smaller root cell diameter in *kai2* mutants may cause tissue tensions leading to skewing [29]. We also observed that *kai2* mutants in both the Col-0 and Ler background had thinner primary roots than wild-type. Quantification of root diameter at 2.5 mm above the root tip confirmed that the primary roots of *kai2* and *max2* mutants but not of the *d14* mutant are thinner than those of the wild type (Fig 4D, S4D Fig). This indicates that the regulation of root thickness is specific to KL signalling. However, we could genetically separate the thin root diameter from the skewing and waving phenotypes because the root diameter phenotype of *max2* could be suppressed by *smax1* without altering the waving phenotypes. Conversely, the *max2* root diameter phenotype could not be suppressed by *smxl2* alone, but *smxl2* was sufficient to suppress the skewing phenotype (Fig 5A and 5C; S5A and S5C Fig). Thus, decreased root diameter is unlikely to cause the skewing and waving phenotypes in *kai2* and *max2* as previously suggested [29].

### KAI2 regulates skewing and waving through SMAX1/SMXL2

The mechanism by which KAI2 regulates root skewing has been proposed to include the non-canonical degradation of SMXL678 [29]. We tested this important hypothesis in more detail, by using different combinations of *smxl* alleles. We observed that, for skewing, *smax1* or *smxl2* were both independently sufficient to suppress the *max2* phenotype (Fig 5A and 5B, S1 Table), indicating that skewing may be very sensitive to the stoichiometry of SMXL proteins or that SMAX1 and SMXL2 act in different cells. *smax1* and *smxl2* could not suppress the *max2* waving phenotype individually, but in combination they were able to completely suppress this phenotype (Fig 5C and 5D, S1 Table), indicating that SMAX1 and SMXL2 act redundantly to promote waving. These results are thus consistent with *SMAX1* and *SMXL2* acting genetically downstream of KAI2 and MAX2 to regulate root growth patterns. Notably, the effect of *kai2*, *smax1* and *smxl2* on skewing was consistent between plants grown in Munich [M] and Leeds [L].

Consistent with the results of [29], we observed a reduction in skewing in *smxl678 max2-1* relative to *max2-1* in plants grown in Munich [M] (Fig 5E). However, this was not the case in Leeds [L], where root skewing was often increased in *smxl678* relative to wild-type, and in which there was an additive increase in skewing in *smxl678 max2-1* (Fig 5G). We also did not observe any suppression of the *max2-1* waving phenotype in *smxl678* (Fig 5F). Thus, our analysis of *smxl678* mutants indicates that SMXL678 proteins likely do not act downstream of KAI2/MAX2 in the regulation of root growth patterns, but rather, that SMXL678 regulates skewing in parallel to the KAI2-SMAX1/SMXL2 pathway.

### SMAX1, SMXL2 as well as SMXL678 regulate lateral root density

**Fig 6:**
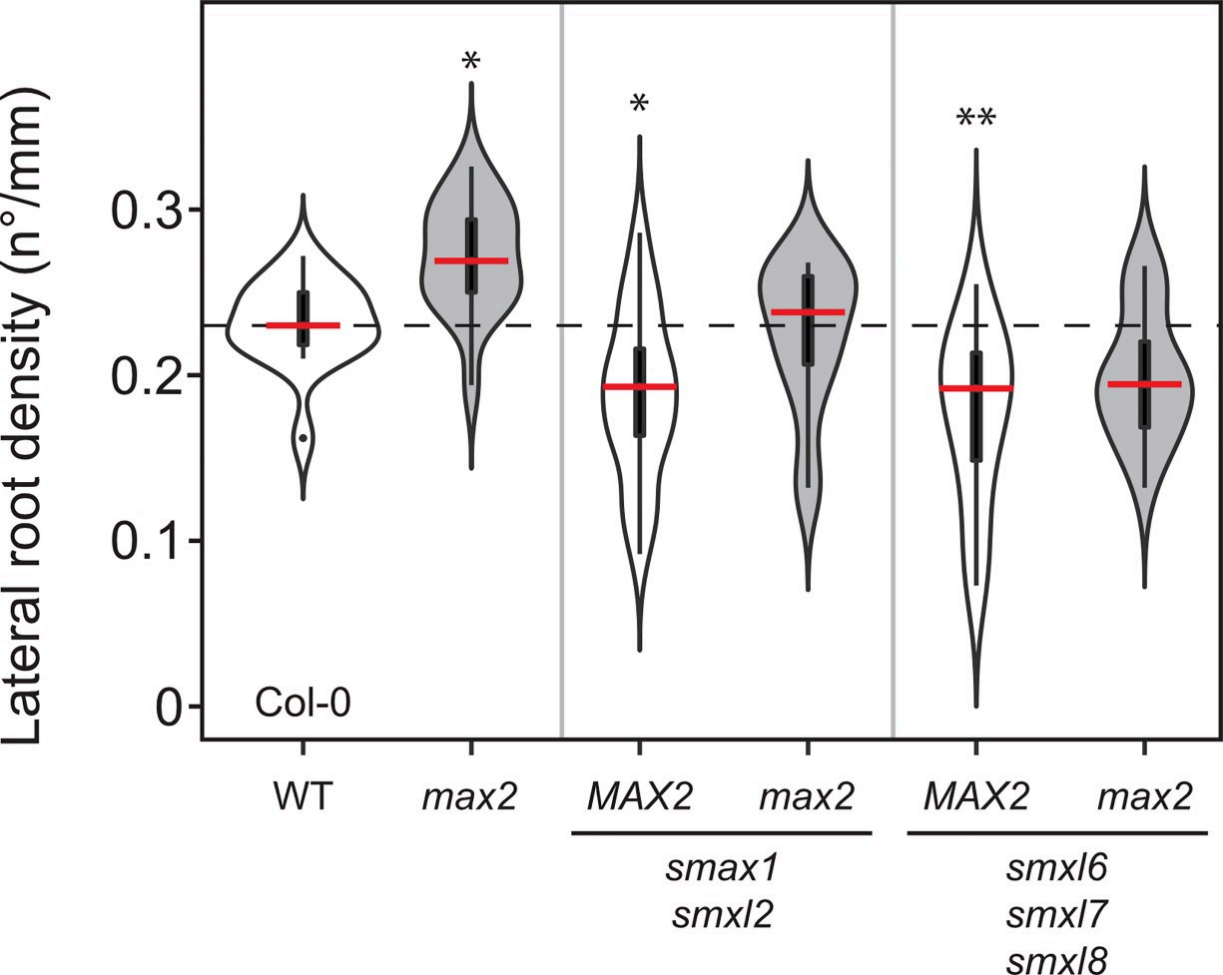
Lateral root density is regulated by both SMAX1/SMXL2 and SMXL678. Lateral root density in Col-0 wild type and the indicated genotypes (the mutant alleles are *max2-1*, *smax1-2*, *smxl2-1*, *smxl6-4*, *smxl7-3* and *smxl8-1*). The outline of the violin plots represents the probability of the kernel density. Black boxes represent interquartile ranges (IQR), with the red horizontal line representing the median; whiskers extend to the highest and lowest data point but no more than ±1.5 times the IQR from the box; outliers are plotted individually. Asterisks indicate a significant difference with wild type (ANOVA, posthoc Dunnett´s test comparing to wild-type, F_5,90_ = 10.62, n = 10–17; *p ≤ 0 .05, **p ≤ 0.01, ***p ≤ 0.001).

Previous results showed that the *max2* LRD phenotype was suppressed in a *smxl678* background but not in a *smax1* background [27], suggesting that the *max2* LRD phenotype arises solely from excess SMXL678 protein accumulation. Since our results show that both D14 and KAI2 regulate LRD, this would again imply non-canonical regulation of SMXL678 by KAI2. To again test this hypothesis, we re-examined the regulation of LRD using more recently-available *smax1 smxl2* double mutants [35]. We found that *smax1 smxl2* was as efficient in reducing LRD of *max2* as *smxl678* (Fig 6). However, consistent with a role of both SL and KL signalling in regulating LRD neither *smax1 smxl2* nor *smxl678* appeared to be completely epistatic to *max2* (Fig 6). The most parsimonious explanation for these results is that the *max2* LRD phenotype arises from the accumulation of both SMAX1/SMXL2 and SMXL678, and that SL and KL signalling act together in the regulation of LR development by their canonical pathways: SL signalling by promoting SMXL678 turnover, and KL signalling by promoting SMAX1 SMXL2 turnover.

### SMAX1 and SMXL2 but not SMXL678 regulate root hair development

**Fig 7:**
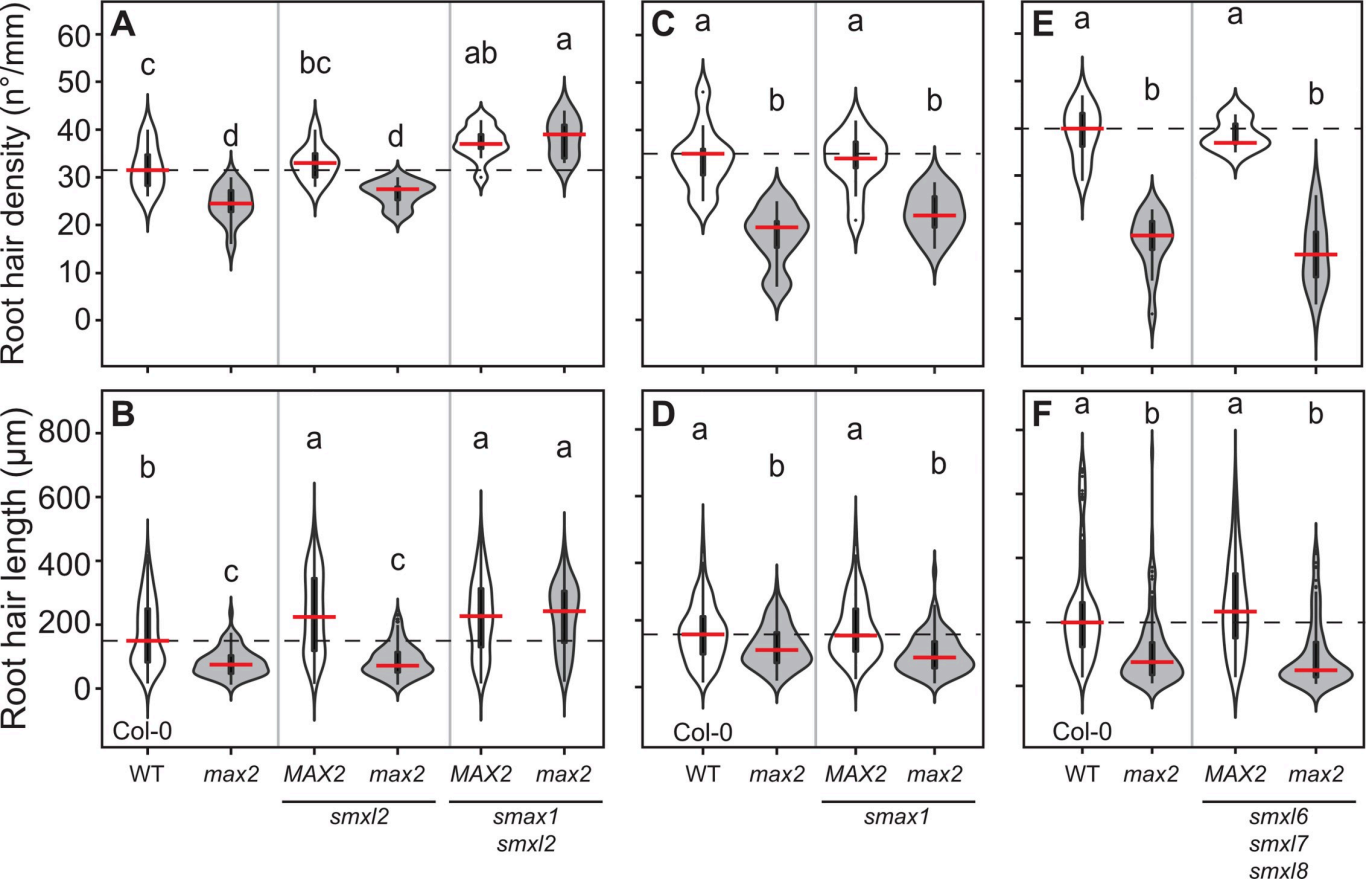
SMAX1 and SMXL2 regulate root hair development. **(A, C, E)** Root hair density and **(B, D, F)** root hair length in Col-0 wild type and the indicated genotypes (the mutant alleles are *max2-1*, *smax1-2*, *smxl2-1*, *smxl6-4*, *smxl7-3* and *smxl8-1*). The outline of the violin plot represents the probability of the kernel density. Black boxes represent interquartile ranges (IQR), with the red horizontal line representing the median; whiskers extend to the highest and lowest data point but no more than ±1.5 times the IQR from the box; outliers are plotted individually. Different letters indicate different statistical groups (ANOVA, posthoc Tukey, p≤ 0.001 **(A)** F_17,385_ = 79.17, n = 9–13 **(B)** F_3,44_ = 67.45, n = 9–11 **(C)** F_3,39_ = 20.33, n = 9–11 **(D)** F_3,615_ = 30.02, n = 9–11 **(E)** F_3,44_ = 67.45, n = 9–15 **(F)** F_3,410_ = 38.66, n = 8–11).

We also assessed, whether regulation of RHD and RHL by KAI2 occurs through canonical or non-canonical signalling. For both RHD and RHL, we found that *smax1 smxl2* have increased RHD and RHL, and are epistatic to *max2-1* in both of these phenotypes. *smxl2* but not *smax1* single mutants display an increased RHL with respect to the wild type, suggesting that SMXL2 may be more important in regulating RHL than SMAX1. Conversely, *smxl678* mutants have no RHD or RHL phenotype, and no effect on the *max2* phenotype (Fig 7A–7F). This is consistent with our observation that *kai2* and not *d14* phenocopies the root hair phenotype of *max2* and that root hair development is regulated by KL signalling under standard conditions.

### The stereoisomers GR24^5DS^ and GR24^*ent*-5DS^ non-specifically enhance root hair development through both D14 and KAI2

**Fig 8:**
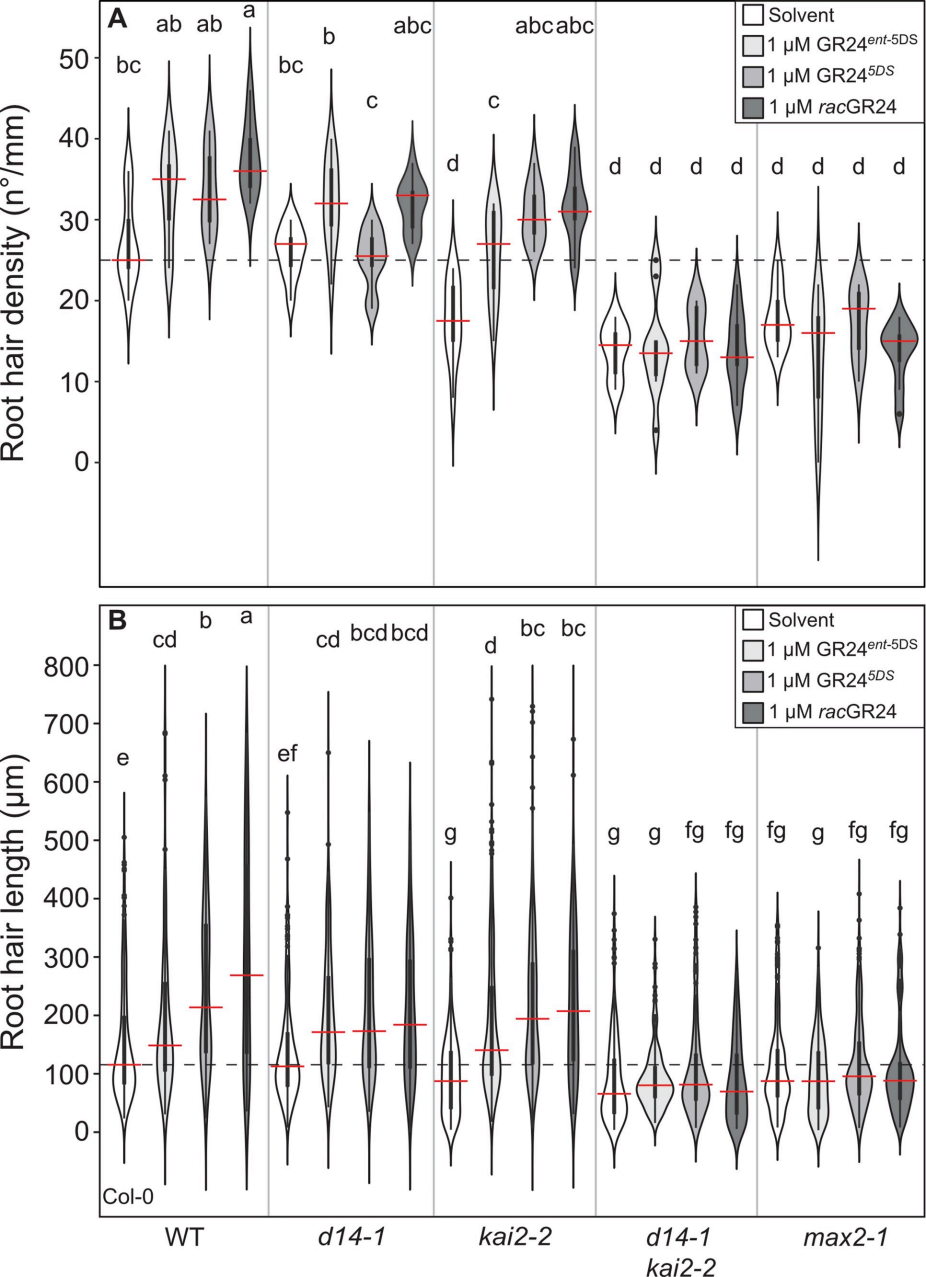
The two GR24 stereoisomers regulate root hair development through both D14 and KAI2. **(A)** Root hair density and **(B)** root hair length of the indicated genotypes treated with solvent (acetone), 1 μM μM GR24^*ent-*5DS^, 1 μM GR24^5DS^ or 1 μM rac-GR24. The outline of the violin plot represents the probability of the kernel density. Black boxes represent interquartile ranges (IQR), with the red horizontal line representing the median; whiskers extend to the highest and lowest data point but no more than ±1.5 times the IQR from the box; outliers are plotted individually. Different letters indicate different statistical groups (ANOVA, posthoc Tukey, n = 8–11 **(A)** F_19,3740_ = 1.983; p≤ 0.01 **(B)** F_19,3740_ = 57.83, p≤ 0.001).

As a final test for non-canonical signalling in root development, we examined ligand-receptor interactions, using the easily scorable, karrikin-responsive root hair phenotypes as a system. Exogenous application of *rac*-GR24 was previously shown to promote root hair elongation [7, 42]. In light of the effects of KAI2 mutations on root hair development, we hypothesized that *rac*-GR24, and in particular the GR24^*ent*-5DS^ stereoisomer, would modulate RHD and RHL, in a manner dependent on KAI2 [34]. Similar to KAR_2_, *rac*-GR24 treatment increased both RHD and RHL in Col-0 (Fig 8A and 8B), and this effect was dependent on *MAX2* as previously reported [7, 42]. However, unexpectedly, it was independent of *KAI2*, suggesting that *rac*-GR24 might promote RHD and RHL via D14 (Fig 8A and 8B). We assessed this in detail and quantified RHD and RHL after treatment with the pure stereoisomers GR24^5DS^ and GR24^*ent*-5DS^, which are thought to specifically activate D14 and KAI2, respectively [34]. We observed that both GR24^5DS^ and GR24^*ent*-5DS^ promote RHD and RHL in the wild-type, but their effects in *d14* and *kai2* mutants were intriguingly divergent from expectations. In *d14-1*, only GR24^*ent*-5DS^ promotes RHD (as expected), but both GR24^5DS^ and GR24^*ent*-5DS^ promote RHL to a similar degree in *kai2*, suggesting that both can be perceived by KAI2 to promote RHL (Fig 3A and 3B). Furthermore, both stereoisomers cause increased RHD and RHL in *kai2-2*, although the ‘canonical’ D14 ligand GR24^5DS^ has a significantly stronger effect than GR24^*ent*-5DS^ (Fig 8A and 8B). Neither stereoisomer promoted RHD and RHL in the *d14-1 kai2-2* double and *max2-1* mutants (Fig 8), confirming that no additional unknown receptor is involved in the response to *rac*-GR24. The first major implication of these results is that D14 can act to promote root hair development, when stimulated with ligand, even if that is not the standard function of D14 (Fig 2). The second major implication is that in roots, contrary to previous suggestions for the regulation of Arabidopsis hypocotyl elongation [34], D14 can perceive GR24^*ent*-5DS^ ligands when KAI2 is absent, and KAI2 can perceive GR24^5DS^ ligands when D14 is absent.

Since these results are unexpected we wondered whether the GR24 stereoisomers we used are really pure and determined their purity by nuclear magnetic resonance (NMR), circular dichroism (CD) spectroscopy and polarimetry (S6 Fig). Both ^1^H-NMR, ^13^C-NMR and CD as well as rotation values determined by means of polarimetric measurements confirmed the purity of the compounds and recapitulated previously published NMR- and CD-spectra for (+)-5-Desoxystrigol and (–)-*ent*-5-Desoxystrigol [46, 47]. Since the stereoisomers are pure, we conclude that they do not specifically act through KAI2 or D14 but that both molecules can bind to and trigger both receptors in the context of root hair development.

Previous Arabidopsis hypocotyl elongation assays suggested specific roles of GR24^5DS^ and GR24^*ent*-5DS^ in triggering D14- vs KAI2-mediated signalling respectively because GR24^5DS^ suppressed hypocotyl elongation specifically in *kai2* mutants and GR24^*ent*-5DS^ in *d14* mutants [34]. We re-examined the effects of the GR24 stereoisomers on hypocotyl elongation (S7 Fig). Similar to root hair elongation and contrary to a previous report [34] the *d14-1* mutant responds equally to GR24^5DS^ and GR24^*ent*-5DS^ with a decrease in hypocotyl growth, showing that in the hypocotyl KAI2 can mediate responses to both molecules. The *kai2-1* mutant also responds to both molecules but to a lesser extent to GR24^*ent*-5DS^, suggesting together with the above results that D14 is more effective in mediating responses to its previously suggested ligand GR24^5DS^ than to GR24^*ent*-5DS^ [34]. Similar to root hair development, the *d14-1 kai2-2* double mutant and the *max2-1* mutant do not respond to any molecule in this assay, confirming that in the hypocotyl response to the GR24 stereoisomers no additional receptor is involved. In summary, we show that GR24^5DS^ and GR24^*ent*-5DS^ can activate both signalling through KAI2 and through D14 in the regulation of RHL as well as hypocotyl elongation.

## Discussion

**Fig 9:**
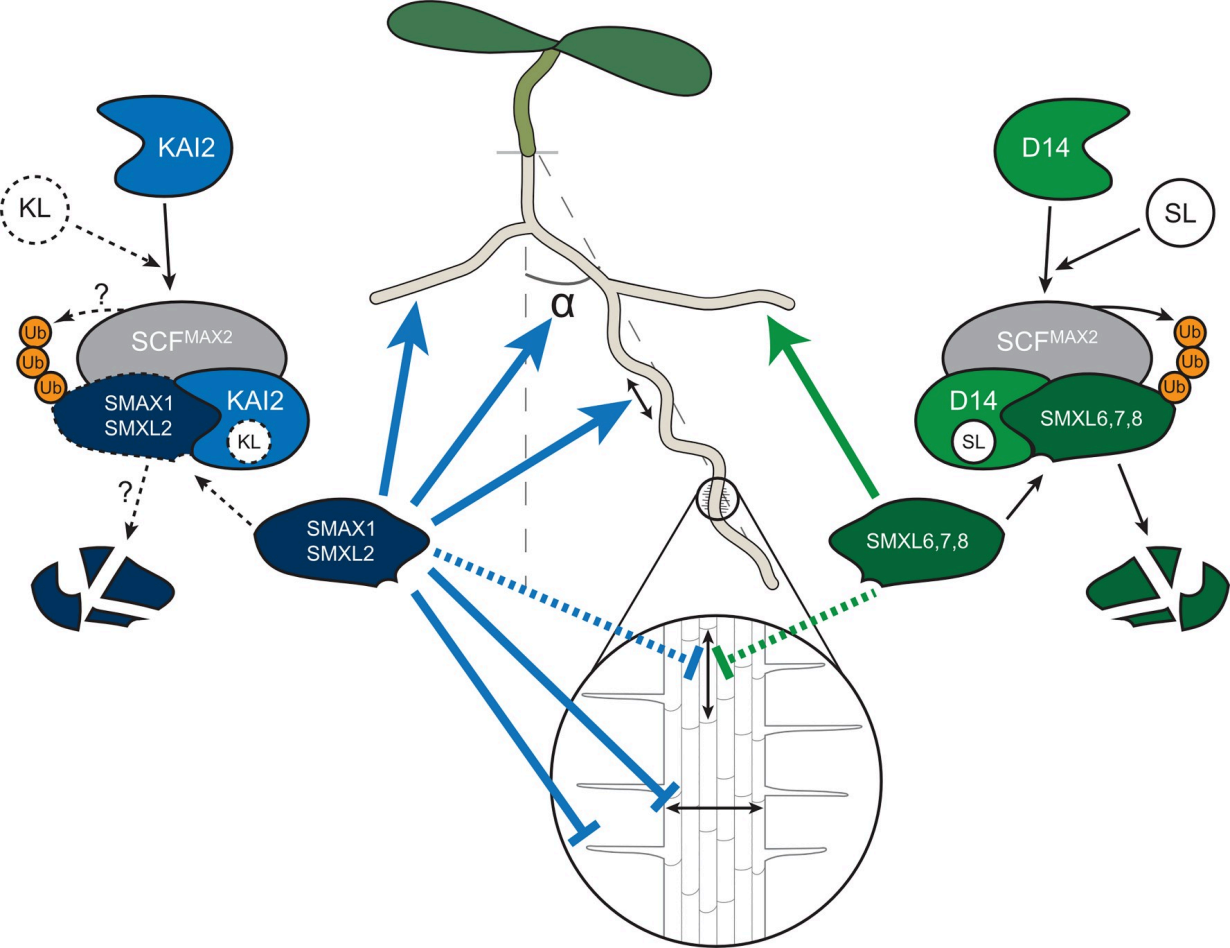
Model for KL and SL signalling regulating Arabidopsis root development. SL and KL signalling act through the proteasomal degradation of SMXLs in Arabidopsis roots. As in the shoot [30] SMAX1 and SMXL2 are targets of KL perception, while SMXL6,7,8 are targets of SL perception. SMAX1 represses root diameter. SMAX1 and SMXL2 repress root hair development and promote root skewing and root straightness. SMAX1, SMXL2, and SMXL6,7,8 promote lateral root development and probably repress cell elongation. Relationships, which are inferred from circumstantial evidence (or for KL signalling from SL signalling) are shown by a dashed arrow or frame.

Root systems flexibly adapt their architecture and morphology to heterogeneous soil environments and to the physiological needs of the plant. A network of plant hormone signalling pathways is essential for translating environmental signals and physiological states into developmental outputs [48]. Strigolactones (SLs) have been assumed to play an important role in modulating root development [7–9]. Here we demonstrate that under standard growth conditions KL signalling plays a much larger role than SL signaling in shaping root and root hair development (Fig 9).

### KL signalling regulates lateral root density together with SL signalling

Previous reports showed increased LRD in *max2* and suppression of lateral root emergence by *rac*-GR24 [8, 9]. Our study indicates that these effects are mediated through both the KAI2 and D14 signalling pathways, in an additive manner. We observed that lateral root density (LRD) is consistently higher in *kai2* mutants than wild type (particularly at earlier time points). We found SL biosynthesis and perception mutants also displayed subtle changes in root architectural parameters, such as primary root length (PRL) and LRD. In a range of experiments with SL mutants, we either observed strongly decreased PRL or strongly increased LRD, but not both phenotypes together. This suggests that the effects of SL signalling on PRL or LRD are to some extent mutually exclusive, and that expression of one phenotype reduces expression of the other, which may explain some of the previous contradictory reports regarding effects of SLs on root development [8, 9]. We also found that the time after germination matters for the LRD phenotypes. Thus, confusion about the role of SLs in LR development may also reflect differences in the physiological timing of observations within experiments. The *d14 kai2* double mutant showed a much larger increase in LRD compared to the single mutants, indicating that both signalling pathways contribute additively to modulating LRD, and that previously reported *max2* phenotypes reflect a lack of both signalling pathways. This is further supported by suppression of the *max2* LRD phenotype by mutants in both the targets of KL signalling (SMAX1/SMXL2) and SL signalling (SMXL678).

### KL signalling is a key regulator of root hair development

A major finding of our work is the important role of KL signalling in root hair development. Root hair density (RHD) and root hair length (RHL) are strongly reduced in *kai2* and *max2* mutants and increased in *smax1 smxl2* mutants, as well as by karrikin treatment of wild type roots. Our results thus present compelling evidence that KL signalling is a key regulator of root hair development. KAI2 being a major regulator of root hair development rather than D14 seems to make sense from an evolutionary point of view. Root hair development and tip growth in Arabidopsis rely on conserved functions and genes that also operate in the development of rhizoids of *Marchantia polymorpha* gametophytes, which appear to be homologous to root hairs [49–51]. *D14* occurs only in genomes of seed plants while *KAI2* is already present in algae [19, 20, 22]. Thus, it is possible that KAI2-SMAX1 module is part of an ancient and conserved pathway regulating tip growth of epidermal cells.

We did not find any impact of *d14* and SL biosynthesis mutants on root hair development in our study. However, we found that D14 signalling can be triggered to promote root hair development, if the correct ligand is present and KAI2 is absent. This is very similar to the hypocotyl, in which D14-mediated SL perception can regulate hypocotyl elongation, but is not actually required to do so [19, 34]. This suggests that there may be a role for D14 signalling in root hair development under certain environmental conditions, when SL levels are very high, for example under phosphate starvation [52]. Previous studies, [53, 54] found a small decrease in RHD of the SL biosynthesis mutant *max4-1*, which could be rescued by adding GR24^5DS^ [54]. This is inconsistent with our observations here, but might reflect differences in the growth conditions used, and indeed these studies used low phosphate media. Thus, further investigation of the role of D14 signalling in environment-dependent root hair development is warranted.

### KL signalling suppresses skewing and waving independently of root cellular parameters

No single signalling pathway for control of root skewing and straightness has been identified, but several studies have exposed different pathways impinging on these root behaviors (reviewed in Roy and Bassham. 2014). The activities of multiple hormones, such as auxin and ethylene, are among the candidates [55, 56]. Here we demonstrate that KL signalling is a novel regulator of root skewing and root straightness. The increased skewing and waving phenotypes of KL perception mutants were found in both the Col-0 and Ler background although Ler shows an intrinsically higher tendency to skew than Col-0. Our results are broadly consistent with the recent report of [29], but our interpretation of the cause of the phenotype differs. Swarbreck et al. [29], speculated that skewing may be caused by increased epidermal cell file rotation and/or smaller root diameter of *kai2* mutants. Under our conditions, we did not observe epidermal cell file rotation in *kai2* and *max2*, but rather shorter epidermal cells. Since both *kai2* and *d14* have a reduced epidermal cell length, but skewing only occurs in *kai2*, we conclude that epidermal cell length is not related to skewing. Interestingly, in the experiments in which epidermal cell length was inspected, PRL was not significantly altered. This implies that a compensatory increase in epidermal cell division must occur in both the KL and SL perception mutants, which would be consistent with increased cell division in the primary root meristem. Alternatively, the epidermal cell length may differ among different root zones thus compensating for the shorter epidermal cell length in the zone 2–3 mm above the root tip. We also show that the reduced root diameter of KL perception mutants does not cause either skewing or waving since *smax1* alone suppresses the root diameter but not the waving phenotype of *max2*, and *smxl2* suppresses the *max2* skewing but not the root diameter phenotype.

### KL and SL signalling in the root employ the canonical receptor-target pairs

We have previously highlighted some phenotypic characteristics suggesting that KL and SL signalling in the root might not act through the canonical KAI2-SMAX1 and D14-SMXL678 receptor-target pairs [10]. The main reason for this suggestion was that *max2* mutants had stronger LRD phenotypes than SL biosynthesis mutants [7–9], which suggested that KAI2 regulates lateral root emergence rather than or in addition to D14, while mutations of the genes encoding the canonical SL signalling targets SMXL678 were able to completely suppress the *max2* LRD phenotype with *smax1* being unable to do so [10, 27]. Similarly, Swarbreck et al. [29] suggested that non-canonical signalling may occur in skewing responses, since *smxl678* mutants can completely suppress the *max2* skewing phenotype, which arises solely through lack of KAI2 signalling.

We have now robustly tested this hypothesis, and find no evidence for non-canonical KL and SL signalling in roots under our growth conditions. Using *smax1 smxl2* double mutants, we show that every effect of loss of KAI2 activity can be suppressed by loss of SMAX1 and SMXL2 (or only one of the two), and that similarly, all effects of loss of D14 activity can be suppressed by loss of SMXL678. In the case of LRD, we show that *smax1 smxl2* mutants can suppress the phenotype of *max2*, demonstrating that the canonical KL signalling targets are involved in regulating lateral root emergence and that *SMXL2* compensates for the absence of functional *SMAX1* in lateral root development [27]. The suppression of the *max2* LRD phenotype by *smxl678* as well as *smax1 smlx2* is consistent with our observation that both D14 and KAI2 regulate LRD. Thus, the accumulation of both SMAX1/SMXL2 and SMXL678 contributes to *max2* LRD phenotypes and there is no need to invoke non-canonical receptor-target pairs to explain the effects of KAI2 and D14 on LRD.

We also reject the idea that KL signalling regulates skewing through SMXL678 [29]. We find that *smxl2* mutations are sufficient to suppress skewing in *max2*, consistent with canonical KAI2-SMAX1/SMXL2 signalling acting in this response. It is certainly interesting that *smxl678* mutants suppress skewing of *max2* under some conditions, which does not reflect any known effect of D14 signalling. However, we show that this phenotype is highly variable, and under our growth conditions in Leeds, *smxl678* mutants actually increased root skewing additively with *max2*. Thus, although SMXL678 can certainly regulate skewing, this appears to be unrelated to the clearly defined and consistent effect of KL signalling on skewing. In fact, it appears consistent with the observation that *rac-*GR24 treatment–which stimulates SMXL678 degradation–causes an increase in root skewing in the wild type [29]. The location-dependent skewing behaviour of *smxl678* mutants suggests that the role of SMXL678 in skewing may strongly depend on environmental conditions, and it will be interesting to identify the mechanisms underlying this phenomenon in the future.

The case is even more clear-cut for RHL, RHD, root straightness and root diameter, for which only *kai2* and *max2* mutants show a phenotypic difference to wild type, and which can only be suppressed by mutating *SMAX1* and *SMXL2*. Interestingly, the *smxl2* mutant alone has longer root hairs than wild-type showing for the first time a phenotype in which SMXL2 plays a more important role than SMAX1, although it is alone not sufficient to suppress the *max2* phenotype. In the case of root diameter, mutation of *SMAX1* is sufficient to suppress the *max2* phenotypes (S2 Table). This partial redundancy of SMAX1 and SMXL2 is also seen in seed germination, hypocotyl growth and leaf shape [27, 35]. This likely arises from different expression patterns of the two genes: in tissues where only one of the two proteins is expressed, removing this one is sufficient to suppress the phenotype. Conversely, in the case of skewing, removing either *SMAX1* or *SMXL2* alone suffices to suppress the *max2* phenotype (S1 Table), suggesting that skewing is particularly sensitive to SMAX1/SMXL2 levels or stoichiometry or that SMAX1 or SMXL2 regulate skewing in different tissues.

### D14 and KAI2 are not completely ligand stereo-specific

In contrast to the lack of evidence for non-canonical receptor-target interactions, we uncovered unexpected evidence for non-canonical receptor ligand interactions in the context of root development. The two stereoisomers of *rac*-GR24, GR24^5DS^ and GR24^*ent*5DS^ have been suggested to specifically activate D14 and KAI2 respectively in the regulation of hypocotyl growth [34]; and GR24^*ent*5DS^ showed only a very low efficiency in inhibiting shoot branching in Arabidopsis and rice [34, 57]. However, our study shows that there is very little specificity of the two receptors for the two stereoisomers, as both *d14* and *kai2* mutants respond to both with increased RHL and even with decreased hypocotyl elongation. This result is strengthened by confirming the purity of the employed compounds via NMR and CD. It has been shown by differential scanning fluorimetry (DSF) *in vitro* that D14 can bind both GR24^5DS^ and GR24^*ent*-5DS^ but KAI2 only bound GR24^*ent*-5DS^ [31]. However, the situation *in vivo* may be different and binding of both ligands to both α/β hydrolase receptors D14 and KAI2 may be stabilized through receptor protein complexes. Although binding of the ‘wrong’ stereoisomer to the α/β hydrolase receptor may be less efficient than binding of the ‘correct’ one, it may suffice to trigger developmental responses, which are very sensitive to removal of SMXL proteins, or which may require additional interaction partners in the receptor complex that stabilize the complex in presence of the hypo-specific ligand. Independent of the mechanism, our results show that GR24^5DS^ and GR24^*ent*-5DS^ cannot safely be used to specifically trigger D14 and KAI2-mediated signalling, respectively. This also implies that the community urgently needs an affordable synthetic SL that triggers D14 in a highly specific manner.

### Regulation of root development by KAI2 and D14 signalling

Overall our results show that KL signaling and therefore SMAX1 and SMXL2 play an important role in controlling root architecture and root hair development (Fig 9). However, some traits such as LRD and epidermal cell length are regulated by both SMAX1/SMXL2 and SMXL678. Key challenges for future studies will be to understand how exactly SMXL proteins regulate root architecture. Ruyter-Spira et al. [8] previously suggested that the impact of SLs on root development might be best understood as a reflection of their effect on the auxin landscape, and we hypothesize that this may also be the case for KAI2 signalling. Most of the traits we have examined are known to be regulated by auxin, and SL signalling in the shoot is known to modulate auxin transport by regulating PIN protein abundance [27, 58]. Thus, it is very possible that the KAI2-SMAX1/SMXL2 and D14-SMXL678 pairs regulate the auxin landscape of the root, for example by controlling the abundance of auxin transport proteins. Such a scenario might underlie the variability in phenotypes observed in the mutants in our study (for instance, the strong variation in *smxl678* skewing phenotype), since environmental parameters such as light or temperature are known to affect endogenous auxin levels [59, 60].

We do not currently know enough about the upstream inputs into the KL signalling pathway to understand the aetiology of KAI2-induced root development, but undoubtedly the phenotypes described here will provide important clues and tools in this regard. SL production increases in several plant species upon phosphate starvation [12, 61–63] and the effect of SL biosynthesis on root architecture was suggested to depend on the sucrose level in the medium and thus on the carbon-status of the plants [8], but it is yet unknown whether KL signalling is also influenced by mineral nutrient levels. However, expression of KAI2 does respond to light conditions, and thus KL signalling could potentially integrate light cues into root development [64]. Indeed, it is likely that both signalling pathways are influenced by multiple abiotic and perhaps biotic stimuli, and it will be exciting to learn how SL and KL signalling tune root development to environmental conditions.

## Materials and methods

### Plant material

*Arabidopsis thaliana* genotypes were in Columbia-0 (Col-0) or Landsberg *erecta* (Ler) parental backgrounds. The following mutants were used: Ler: *max2-8* [18], *kai2-1*, *kai2-2* [18], Col-0: *kai2-2* [28], *max3-9* [65], *max4-5*, *d14-1 kai2-2* [66], *d14-1* [19], *max1-1*, *max2-1*, *max2-2* [67], *smax1-2*, *max2-1 smax1-2* [37], *smax1-2 smxl2-1*, *max2-1 smax1-2 smxl2-1* [35], *smxl6-4 smxl7-3 smxl8-1*, *max2-1 smxl6-4 smxl7-3 smxl8-1* [27].

### Plant growth conditions

For analysis of root growth, *Arabidopsis thaliana* seeds were grown in axenic conditions on 12x12cm square plates containing 60 ml agar-solidified medium. Seed were surface sterilized either by vapour sterilization, or by washing with 1 ml of 70% (v/v) ethanol and 0.05% (v/v) Triton X-100 with gentle mixing by inversion for 6 minutes at room temperature, followed by 1 wash with 96% ethanol and 5 washes with sterile distilled water. For primary root length and lateral root density plants were grown in Cambridge and Leeds on plates containing ATS medium [68] supplemented with 1% sucrose (w/v) and solidified with 0.8% ATS. For measurements of skewing, waving, cell length, root diameter, root hair density and root hair length, seedlings were grown in Munich on plates containing 0.5X Murashige & Skoog medium, pH5.8 (½ MS) (Duchefa, Netherlands), supplemented with 1% sucrose and solidified with 1.5% agar. Plates were stratified at 4°C for 2–3 days in the dark, and then transferred to a growth cabinet under controlled conditions at 22°C, 16-h/8-h light/dark cycle (intensity ~120 μmol m^-2^ s^-1^). Unless otherwise indicated, the plates were placed vertically.

### Phytohormone treatments

*rac*-GR24 was purchased from Chiralix (Nijmegen, The Netherlands), GR24^*ent5DS*^ and GR24^*5DS*^ from Strigolab (Turin, Italy), and KAR_2_ from Olchemim (Olomouc, Czech Republic). For treatment with *rac*-GR24, GR24^*ent5DS*^ or GR24^*5DS*^, 1 mM stock solutions were prepared in 100% acetone. KAR_2_ was dissolved in 70% methanol for the preparation of 1 mM stock. The volume required to reach the final concentration of these different stock solutions was added to molten media prior to pouring Petri dishes. In each experiment, an equivalent volume of solvent was added to Petri dishes for untreated controls.

### Primary and lateral root quantification

For quantification of primary root length and lateral root number, seedlings were grown as described above in Cambridge and Leeds for 10 days post germination (dpg). This allowed for the emergence of lateral roots sufficient for quantification in wild-type seedlings. A dissecting microscope was used to count emerged lateral roots in each root system, and images of the plates were then taken using a flatbed scanner. Primary root length was quantified using Image J. Separate experiments were primarily used to assess root skewing (see below), but root skewing angles were also measured from these images generated in these experiments.

### Root skewing and straightness assay

The root slanting assay was modified from the method described by [69]. Arabidopsis seedlings were grown in Munich under the conditions described above (except for Fig 8G for which plants were grown in Leeds). Images were taken 5 days post germination (dpg) using an Epson Perfection V800 Pro Scanner. Images were analysed using the Simple Neurite Tracer plug-in of Fiji (https://imagej.net/Fiji/Downloads) to determine the following parameters as illustrated in Fig 4; root length (L), ratio of the straight line between the hypocotyl-root junction and the root tip (Lc), and vertical axis (Ly). These measurements were taken from at least 60 individual roots per genotype and used to calculate the root skewing angle (α) and root straightness (Lc/L) as previously described [70, 71].

### Determination of root hair density, length and position

Root hair growth was examined in Munich on the same *Arabidopsis* roots, which were used for determining root skewing and straightness. Images were taken at 2 mm from the root tip of a minimum of 8 roots per genotype and treatment with a Leica DM6 B microscope equipped with a Leica DFC9000 GT camera. The number of root hairs was determined by counting the root hairs between 2 and 3 mm from the root tip on each root, and root hair length was measured for 10–18 different root hairs per root using Fiji. The root hair position was determined following the method described by [72] for 5–15 root hairs per root and a minimum of 8 roots per genotype.

### Root diameter and cell length analysis

Using the same images as for root hair quantification, root diameter, root cell length and number of cells were analysed in Munich using Fiji. Root diameter was measured at 2.5 mm from the root tip. The number of cells was defined as the number of epidermal cells that crossed a 1-mm-long straight line drawn between 2 to 3 mm from the root tip. Root cell length was measured for at least 10 different epidermal cells per individual root in a minimum of 10 roots per genotype, between 2 to 3 mm from the root tip.

### Determination of purity of GR24 stereoisomers

#### Chemicals

The following compounds were obtained commercially from the sources given in parentheses: formic acid, chloroform (HPLC grade) (Merck, Darmstadt, Germany); acetonitrile (MS grade, J. T. Baker, Deventer, Netherlands); (CD_3_)_2_CO was obtained from Euriso-Top (Gif-Sur-Yvette, France). Water for UHPLC separation was purified by means of a Milli-Q water advantage A 10 water system (Millipore, Molsheim, France).

#### General experimental procedures

^1^H NMR experiments were performed on an Avance III 400 MHz spectrometer with a BBI probe (Bruker, Rheinstetten, Germany) at 298 K. (CD_3_)_2_CO was used as solvent and chemical shifts are reported in parts per million, relative to solvent signal: ^1^H NMR: 2.05 ppm and ^13^C NMR: 29.84 ppm. Data processing was performed by using Topspin software (version 2.1; Bruker) as well as MestReNova software (version 5.2.3; Mestrelab Research, Santiago de Compostella, Spain). For circular dichroism (CD) spectroscopy, sample solutions of compounds were analysed by means of a Jasco J-810 spectropolarimeter (Hachioji, Japan). High-resolution mass spectra were measured on a TripleTOF 6600 mass spectrometer (Sciex, Darmstadt, Germany) equipped with a DuoSpray source (Sciex), running in ESI positive mode, connected to a Nexera X2 UHPLC (Shimadzu, Duisburg, Germany), consisting of two LC pump systems 30AD, a DGU-20A5 degasser, a SIL-30AC autosampler, a CTO-30A column oven and a CBM-20A controller. Calibration of the mass spectrometer was performed after every 5 samples using a Calibrant Delivery System (Sciex) linked to the APCI probe of the DuoSpray source and either positive or negative APCI Calibration solution (Sciex). Rotation values were determined by means of a P3000 polarimeter (Krüss, Hamburg, Germany). The structures of compound of GR24^5DS^ and GR24^*ent*-5DS^ were characterized, by means of UHPLC-TOF-MS, ^1^H NMR, CD spectroscopy and polarimetric experiments.

**GR24**^**5DS**^: LC-TOF-MS: *m/z* 299.0915 (measured), *m/z* 299.0919 (calcd. for [C_17_H_14_O_5_+H^+^]^+^); ^1^H NMR (400 MHz, (CD_3_)_2_CO): δ/ppm: 7.56 (d, *J* = 2.6 Hz, 1H, H-C(6´)), 7.44 (d, *J* = 7.4 Hz, 1H, H-C(8)), 7.36–7.21 (m, 3H, H-C(5–7)), 6.55 (t, *J* = 1.4 Hz, 1H, H-C(2´)), 5.94 (d, *J* = 7.9 Hz, 1H, H-C(3´)), 4.02–3.93 (m, 1H, H-(3a)), 3.40 (dd, *J* = 16.9, 9.3 Hz, 1H, H-C(4*α*)), 3.08 (dd, *J* = 16.9, 3.3 Hz, 1H, H-C(4*β*)), 1.95 (t, *J* = 1.5 Hz, 3H, H-C(7´)). ^13^C NMR (100 MHz, (CD_3_)_2_CO): δ/ppm: 171.29 (C = O), 171.28 (C = O), 152.73 C(6´), 143.85 C(8a), 143.24 C(3´), 140.55 C(4a), 135.56 C(4´), 130.59 C(5), 128.09 C(7), 127.04 C(8), 126.09 C(6), 113.45 C(3), 102.24 C(2´), 86.25 C(8b), 39.60 C(3a), 37.85 C(4), 10.60 C(7´). CD(20°C; ACN; c = 0.01 mM) λmax (Δε) 262 (–1.7), 230 (25.5) nm. [α]_D_^15^ +420° (CDCl_3_, *c* 0.25 mM) [+436°, [46]].

**GR24**^**ent-5DS**^: LC-TOF-MS: *m/z* 299.0920 (measured), *m/z* 299.0919 (calcd. for [C_17_H_14_O_5_+H^+^]^+^); ^1^H NMR (400 MHz, (CD_3_)_2_CO): δ/ppm: 7.56 (d, *J* = 2.6 Hz, 1H, H-C(6´)), 7.44 (d, *J* = 7.4 Hz, 1H, H-C(8)), 7.36–7.19 (m, 3H, H-C(5–7)), 6.55 (t, *J* = 1.4 Hz, 1H, H-C(2´)), 5.94 (d, *J* = 7.9 Hz, 1H, H-C(3´)), 4.07–3.92 (m, 1H, H-(3a)), 3.40 (dd, *J* = 16.9, 9.3 Hz, 1H, H-C(4*α*)), 3.08 (dd, *J* = 16.9, 3.3 Hz, 1H, H-C(4*β*)), 1.95 (t, *J* = 1.5 Hz, 3H, H-C(7´)). ^13^C NMR (100 MHz, (CD_3_)_2_CO): δ/ppm: 171.29 (C = O), 171.28 (C = O), 152.73 C(6´), 143.85 C(8a), 143.24 C(3´), 140.55 C(4a), 135.56 C(4´), 130.59 C(5), 128.09 C(7), 127.04 C(8), 126.09 C(6), 113.45 C(3), 102.24 C(2´), 86.25 C(8b), 39.60 C(3a), 37.85 C(4), 10.60 C(7´). CD (20°C; ACN; c = 0.01 mM) λmax (Δε) 262 (1.7), 230 (–26.9) nm. [α]_D_^15^–427° (CDCl_3_, *c* 0.25 mM) [–446°, [46]].

#### Purity of both isomers

93–95% (^1^H NMR).

### Statistical analysis

Statistical analyses were performed in R-studio, using one-way Analysis of Variance (ANOVA), followed by Tukey HSD or Dunnett´s post hoc test.

### Accession numbers

Sequence data for the genes mentioned in this article can be found in The Arabidopsis Information Resource (TAIR; https://www.arabidopsis.org) under the following accession numbers: *MAX3*, AT2G44990; *MAX4*, AT4G32810; *MAX1*, AT2G26170; *D14*, AT3G03990; *KAI2*, AT4G37470; *MAX2*, AT2G42620; *SMAX1*, AT5G57710; *SMXL2* AT4G30350; *SMXL6*, AT1G07200; *SMXL7*, AT2G29970; *SMXL8*, AT2G40130.

## Supporting information

S1 Fig Variation in root growth parameters in strigolactone synthesis and perception mutants. Mean primary root lengths (PRL) and mean lateral root densities (LRD) for strigolactone synthesis mutants (*max1-1*, *max3-9*, *max4-5*) and perception mutants (*d14-1*) across 5 different experiments. Values shown are quoted as a percentage, relative to the mean value for the Col-0 wild-type control in the same experiment (set to 100). Shading of cells represents percent below or above the mean of the wild type. Strong reductions in PRL are never accompanied by strong increase in LRD, and strong increases in LRD are never accompanied by strong reductions in PRL.
(TIFF)

S2 Fig KL signaling regulates lateral root density. **(A)** Lateral root density of the indicated genotypes. **(B)** Lateral root density at 6, 8 or 10 days post germination (dpg). The outline of the violin plot represents the probability of the kernel density. Black boxes represent interquartile ranges (IQR), with the red horizontal line representing the median; whiskers extend to the highest and lowest data point but no more than ±1.5 times the IQR from the box; outliers are plotted individually. Percentage numbers indicate the percent significant difference between the median of each indicated genotype and the median of the wild type at the same time point. Different letters indicate different statistical groups **(A)** ANOVA, posthoc Tukey, F_2,79_ = 5.29, n = 24–30, p<0.01. Asterisks indicate a significant difference compared to wild type for each time point. **(B)** ANOVA, post-hoc Dunnett’s tests comparing to wild-type, at each time-point, F_11,239_ = 47.87, n = 14–24; *p ≤ 0.05, **p ≤ 0.01, ***p ≤ 0.001).
(TIFF)

S3 Fig KAR perception mutants respond to tilted agar surface. **(A, D, E)** Root skewing and **(B, F, G)** root straightness of the indicated genotypes. In **(A, B)** plants were grown at a 90° angle. **(D-E)** Plants were grown either at a 90° angle (white violins) or a 45° angle (grey violins) as shown in the diagram in **(C).** The outline of the violin plots represents the probability of the kernel density. Black boxes represent interquartile ranges (IQR), with the red horizontal line representing the median; whiskers extend to the highest and lowest data point but no more than ±1.5 times the IQR from the box; outliers are plotted individually. Different letters indicate different statistical groups (ANOVA, posthoc Tukey, p≤0.001, n > 40 **(A)** F_5,333_ = 5.057 **(B)** F_4,290_ = 7.168 **(D)** F_7,383_ = 5.788 **(E)** F_7,472_ = 12.54 **(F)** F_7,430_ = 25.89 **(G)** F_7,497_ = 18.36).
(TIFF)

S4 Fig KL perception mutants in the Ler background exhibit decreased epidermal cell lengths and root diameter. **(A)** Number of root epidermal cells per mm of the indicated genotypes. **(B)** Images of representative roots between 2 and 3 mm from the root tip from 5-days-old seedlings of the indicated genotypes. Scale bars, 0.1 mm. **(C)** Root cell length and **(D)** and root diameter of the indicated genotypes. The outline of the violin plots represent the probability of the kernel density. Black boxes represent interquartile ranges (IQR), with the red horizontal line representing the median; whiskers extend to the highest and lowest data point but no more than ±1.5 times the IQR from the box; outliers are plotted individually. Different letters indicate different statistical groups (ANOVA, posthoc Tukey, p≤0.001 **(A)** F_2,43_ = 9.58, n = 13–18 **(C)** F_2,191_ = 43.1, n = 10–11 **(D)** F_2,64_ = 77.45, n = 21).
(TIFF)

S5 Fig Regulation of root skewing by KAI2 can be genetically separated from root diameter. **(A, B, C)** Root diameter of Col-0 wild type and the indicated genotypes (the mutant alleles are *max2-1*, *smax1-2*, *smxl2-1*, *smxl6-4*, *smxl7-3* and *smxl8-1*). The outline of the violin plot represents the probability of the kernel density. Black boxes represent interquartile ranges (IQR), with the red horizontal line representing the median; whiskers extend to the highest and lowest data point but no more than ±1.5 times the IQR from the box; outliers are plotted individually. Different letters indicate different statistical groups (ANOVA, posthoc Tukey, p≤0.001, **(A)** F_3,38_ = 15.04, n = 10–11 **(B)** F_3,38_ = 15.04, n = 8–21 **(C)** F_3,47_ = 8.221, n = 10–11).
(TIFF)

S6 Fig Purity evaluation of SL stereoisomers. **(A)** Chemical structures of GR24^5DS^ and GR24^*ent*-5DS^. **(B)** CD spectra of GR24^*5DS*^ and GR24^*ent-*5DS^. **(C)** ^1^H-NMR (400 MHz, 298 K, (CD_3_)_2_CO) of GR24^5DS^. **(D)** ^13^C-NMR (100 MHz, 298 K, (CD_3_)_2_CO) of GR24^*5DS*^. **(E)** ^1^H-NMR (400 MHz, 298 K, (CD_3_)_2_CO) of GR24^*ent*-5DS^. **(F)** ^13^C-NMR (100 MHz, 298 K, (CD_3_)_2_CO) of GR24^*ent*-5DS^. For more information see Materials and Methods.
(TIFF)

S7 Fig GR24 stereoisomers regulate hypocotyl length through D14 and KAI2. Hypocotyl length of the indicated genotypes treated with solvent (acetone), 1 μM μM GR24^*ent-*5DS^, 1 μM GR24^5DS^ or 1 μM *rac*-GR24. The outline of the violin plot represents the probability of the kernel density. Black boxes represent interquartile ranges (IQR), with the red horizontal line representing the median; whiskers extend to the highest and lowest data point but no more than ±1.5 times the IQR from the box; outliers are plotted individually. Different letters indicate different statistical groups (ANOVA, posthoc Tukey, F_2,43_ = 9.58, n = 32–42, p≤0.001).
(TIFF)

S1 Table Summary of effects of *SMXL* mutations on *max2* root phenotypes. (PDF)

S2 Table Raw data for all figures. (XLSX)
