# Supplementary figures and images for: SMAX1/SMXL2 regulate root and root hair development downstream of KAI2-mediated signalling in Arabidopsis
Source: PLoS Genet. 2019 Aug 29;15(8):e1008327. doi: 10.1371/journal.pgen.1008327 (PMC6738646; doi:10.1371/journal.pgen.1008327)

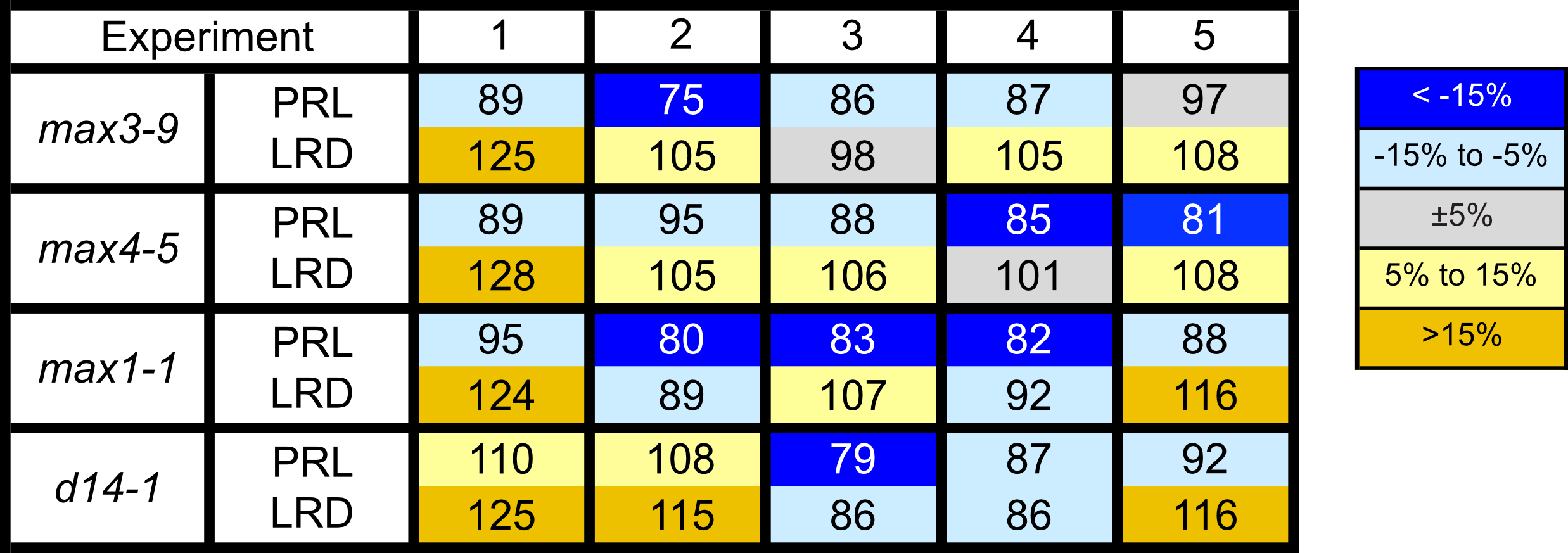

Supplement: S1 Fig — Mean primary root lengths (PRL) and mean lateral root densities (LRD) for strigolactone synthesis mutants (max1-1, max3-9, max4-5) and perception mutants (d14-1) across 5 different experiments. Values shown are quoted as a percentage, relative to the mean value for the Col-0 wild-type control in the same experiment (set to 100). Shading of cells represents percent below or above the mean of the wild type. Strong reductions in PRL are never accompanied by strong increase in LRD, and strong increases in LRD are never accompanied by strong reductions in PRL. (TIFF) [file pgen.1008327.s001.tiff]

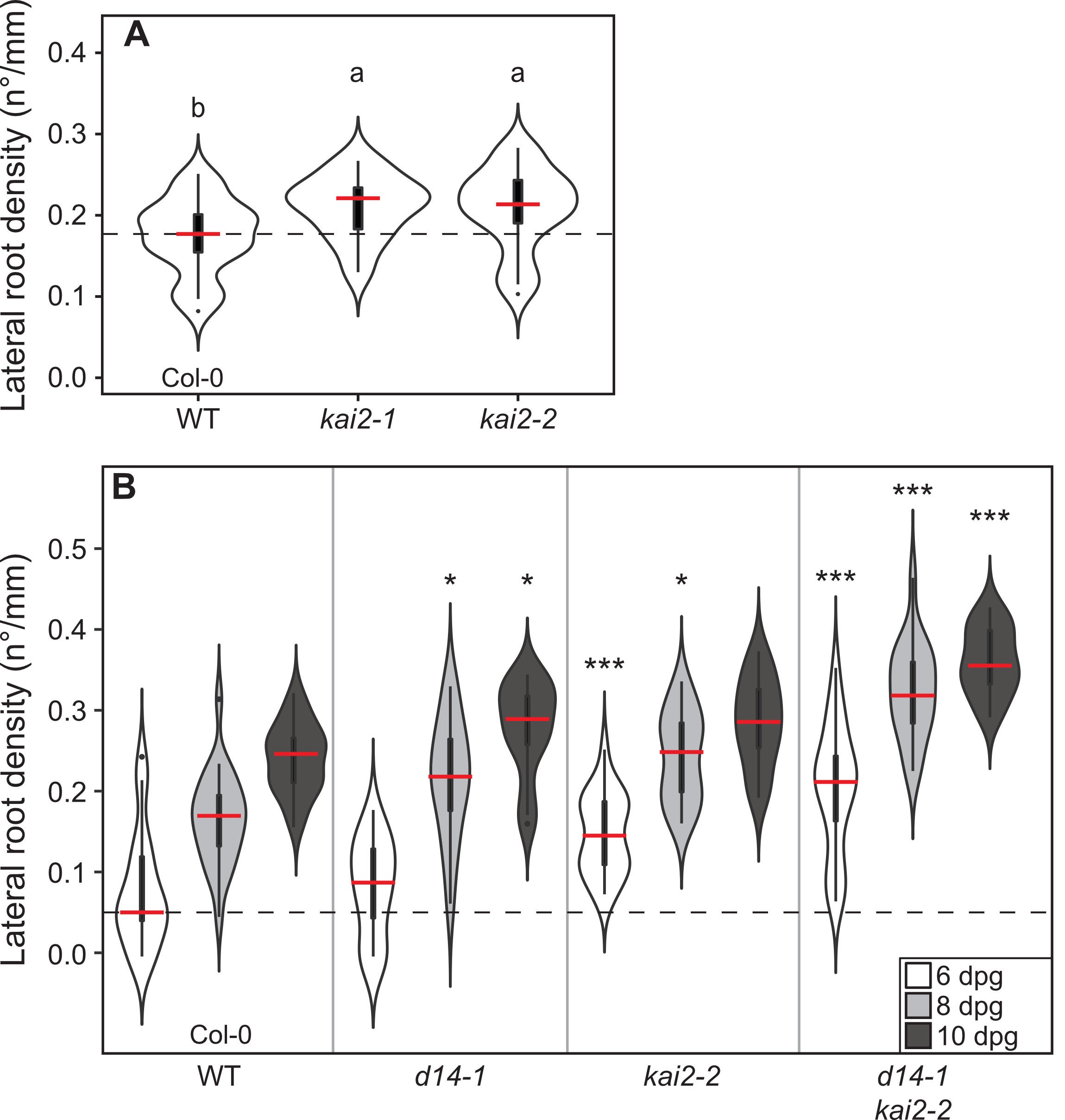

Supplement: S2 Fig — (A) Lateral root density of the indicated genotypes. (B) Lateral root density at 6, 8 or 10 days post germination (dpg). The outline of the violin plot represents the probability of the kernel density. Black boxes represent interquartile ranges (IQR), with the red horizontal line representing the median; whiskers extend to the highest and lowest data point but no more than ±1.5 times the IQR from the box; outliers are plotted individually. Percentage numbers indicate the percent significant difference between the median of each indicated genotype and the median of the wild type at the same time point. Different letters indicate different statistical groups (A) ANOVA, posthoc Tukey, F2,79 = 5.29, n = 24–30, p<0.01. Asterisks indicate a significant difference compared to wild type for each time point. (B) ANOVA, post-hoc Dunnett’s tests comparing to wild-type, at each time-point, F11,239 = 47.87, n = 14–24; *p ≤ 0.05, **p ≤ 0.01, ***p ≤ 0.001). (TIFF) [file pgen.1008327.s002.tiff]

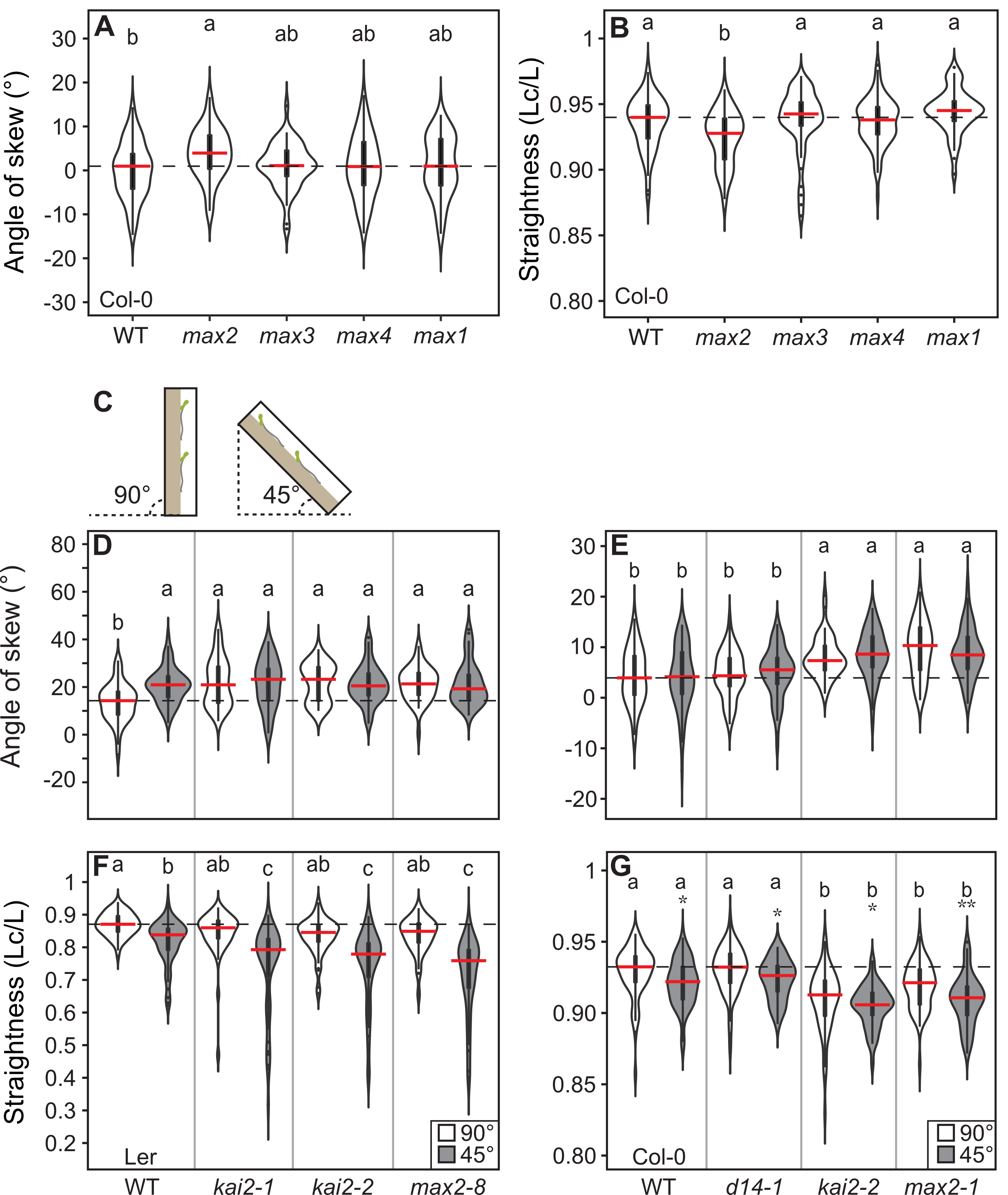

Supplement: S3 Fig — (A, D, E) Root skewing and (B, F, G) root straightness of the indicated genotypes. In (A, B) plants were grown at a 90° angle. (D-E) Plants were grown either at a 90° angle (white violins) or a 45° angle (grey violins) as shown in the diagram in (C). The outline of the violin plots represents the probability of the kernel density. Black boxes represent interquartile ranges (IQR), with the red horizontal line representing the median; whiskers extend to the highest and lowest data point but no more than ±1.5 times the IQR from the box; outliers are plotted individually. Different letters indicate different statistical groups (ANOVA, posthoc Tukey, p≤0.001, n > 40 (A) F5,333 = 5.057 (B) F4,290 = 7.168 (D) F7,383 = 5.788 (E) F7,472 = 12.54 (F) F7,430 = 25.89 (G) F7,497 = 18.36). (TIFF) [file pgen.1008327.s003.tiff]

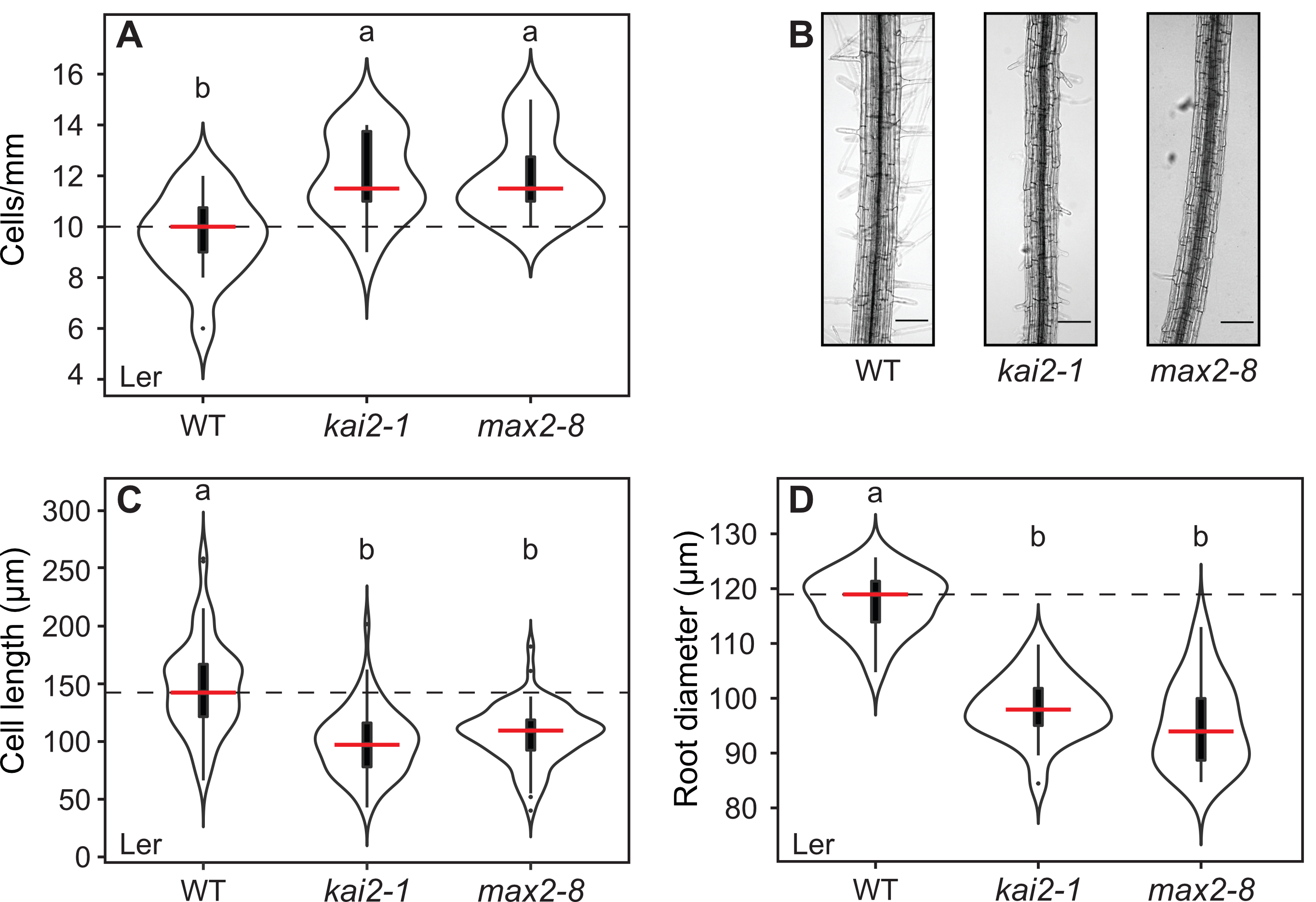

Supplement: S4 Fig — (A) Number of root epidermal cells per mm of the indicated genotypes. (B) Images of representative roots between 2 and 3 mm from the root tip from 5-days-old seedlings of the indicated genotypes. Scale bars, 0.1 mm. (C) Root cell length and (D) and root diameter of the indicated genotypes. The outline of the violin plots represent the probability of the kernel density. Black boxes represent interquartile ranges (IQR), with the red horizontal line representing the median; whiskers extend to the highest and lowest data point but no more than ±1.5 times the IQR from the box; outliers are plotted individually. Different letters indicate different statistical groups (ANOVA, posthoc Tukey, p≤0.001 (A) F2,43 = 9.58, n = 13–18 (C) F2,191 = 43.1, n = 10–11 (D) F2,64 = 77.45, n = 21). (TIFF) [file pgen.1008327.s004.tiff]

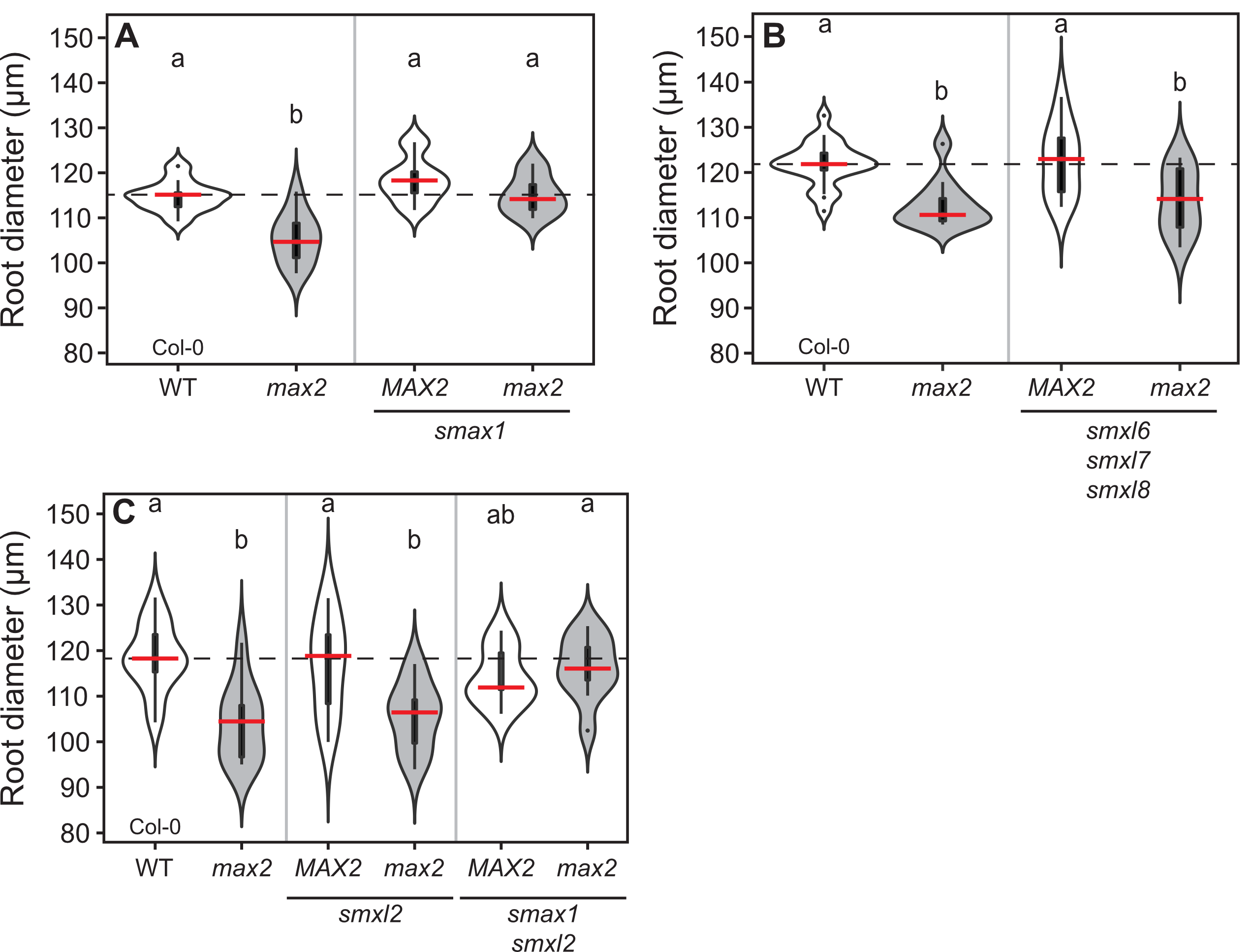

Supplement: S5 Fig — (A, B, C) Root diameter of Col-0 wild type and the indicated genotypes (the mutant alleles are max2-1, smax1-2, smxl2-1, smxl6-4, smxl7-3 and smxl8-1). The outline of the violin plot represents the probability of the kernel density. Black boxes represent interquartile ranges (IQR), with the red horizontal line representing the median; whiskers extend to the highest and lowest data point but no more than ±1.5 times the IQR from the box; outliers are plotted individually. Different letters indicate different statistical groups (ANOVA, posthoc Tukey, p≤0.001, (A) F3,38 = 15.04, n = 10–11 (B) F3,38 = 15.04, n = 8–21 (C) F3,47 = 8.221, n = 10–11). (TIFF) [file pgen.1008327.s005.tiff]

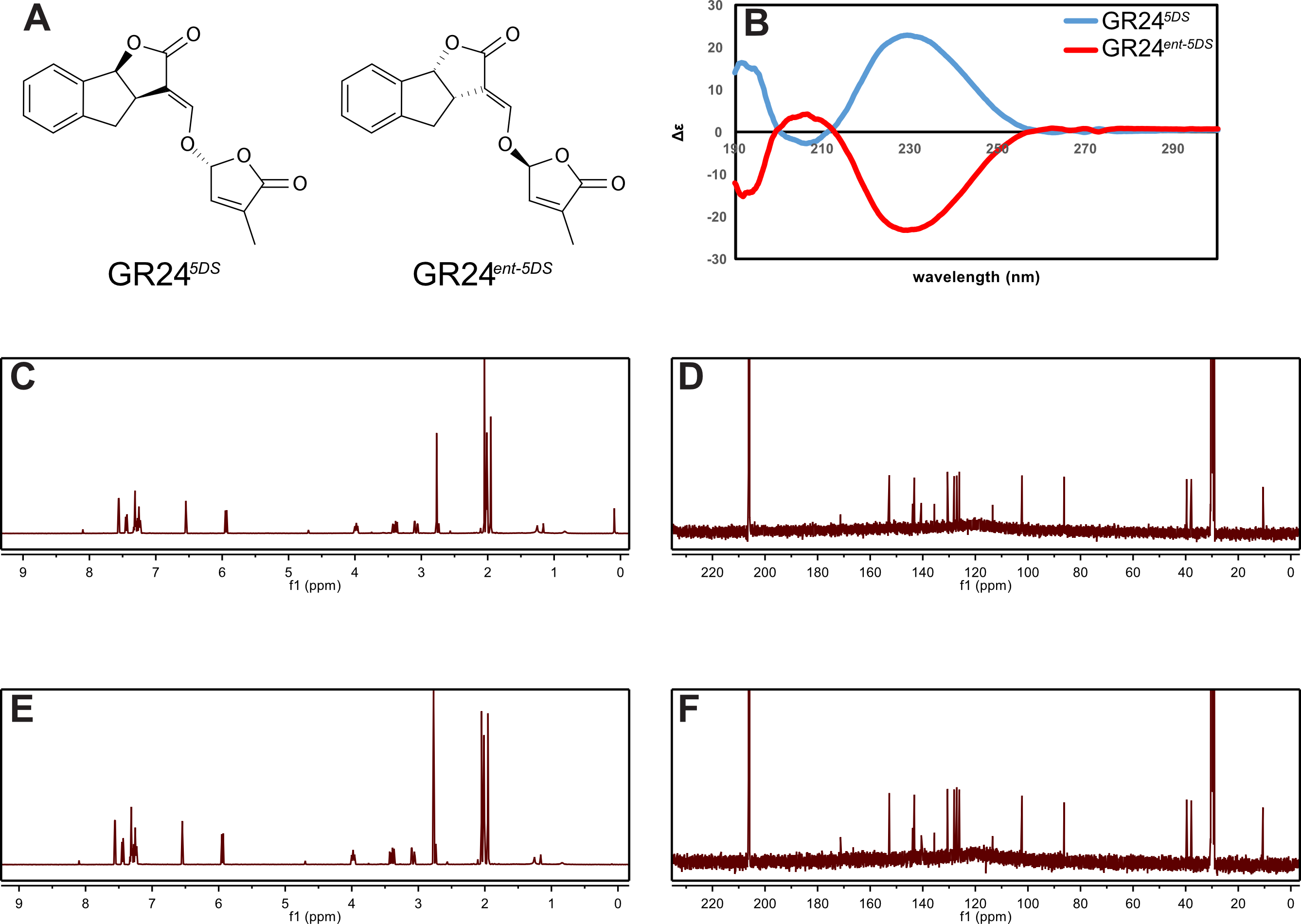

Supplement: S6 Fig — (A) Chemical structures of GR245DS and GR24ent-5DS. (B) CD spectra of GR245DS and GR24ent-5DS. (C) 1H-NMR (400 MHz, 298 K, (CD3)2CO) of GR245DS. (D) 13C-NMR (100 MHz, 298 K, (CD3)2CO) of GR245DS. (E) 1H-NMR (400 MHz, 298 K, (CD3)2CO) of GR24ent-5DS. (F) 13C-NMR (100 MHz, 298 K, (CD3)2CO) of GR24ent-5DS. For more information see Materials and Methods. (TIFF) [file pgen.1008327.s006.tiff]

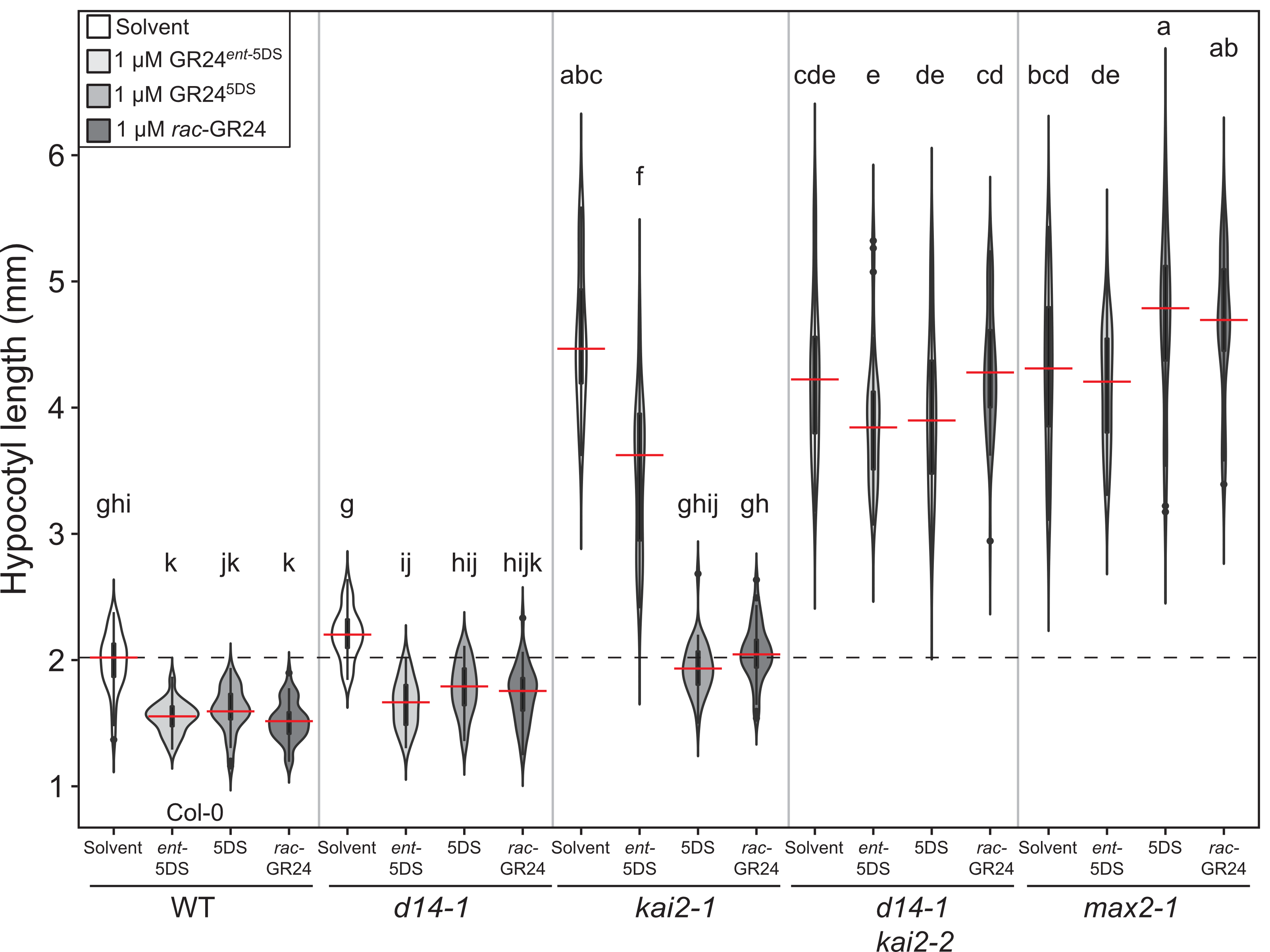

Supplement: S7 Fig — Hypocotyl length of the indicated genotypes treated with solvent (acetone), 1 μM μM GR24ent-5DS, 1 μM GR245DS or 1 μM rac-GR24. The outline of the violin plot represents the probability of the kernel density. Black boxes represent interquartile ranges (IQR), with the red horizontal line representing the median; whiskers extend to the highest and lowest data point but no more than ±1.5 times the IQR from the box; outliers are plotted individually. Different letters indicate different statistical groups (ANOVA, posthoc Tukey, F2,43 = 9.58, n = 32–42, p≤0.001). (TIFF) [file pgen.1008327.s007.tiff]
