# Supplementary material for: SMAX1/SMXL2 regulate root and root hair development downstream of KAI2-mediated signalling in Arabidopsis
Source: PLoS Genet. 2019 Aug 29;15(8):e1008327. doi: 10.1371/journal.pgen.1008327 (PMC6738646; doi:10.1371/journal.pgen.1008327)
Supplement: S1 Table — (PDF) [file pgen.1008327.s008.pdf]

Supplemental Table S1. Summary of effects of *SMXL* mutations on *max2* root phenotypes.

	max2			
	smax1	*smxl2*	*smax1 smxl2*	*smxl6,7,8*
Lateral root density			+	+
Root hair density	-	-	+	-
Root hair length	-	-	+	-
Root skewing	+	+	+	+/-
Root straightness	-	-	+	-
Root diameter	+	-	+	-

\+ Suppression of *max2* phenotypes

\- No suppression of *max2* phenotypes

+/- Opposite effects in Munich (+) and Leeds (-)
